# Recent progress on the biological degradation and solubilization of coal

**DOI:** 10.1007/s10532-025-10175-9

**Published:** 2025-09-15

**Authors:** Lerato M. Sekhohola-Dlamini, Sohail Khan, Bobo Wang, Zhisheng Yu, A. Keith Cowan

**Affiliations:** 1 College of Agriculture and Environmental Sciences, Department of Environmental Sciences, University of South Africa, P.O. Box X6, Florida, Johannesburg, 1710 South Africa; 2 College of Resources and Environment, University of Chinese Academy of Sciences, 19 A Yuquan Road, Beijing, 100049 People’s Republic of China; 3 Institute for Environmental Biotechnology (EBRU), Rhodes University, P.O. Box 94, Makhanda, 6140 South Africa

**Keywords:** Archaea, Bacteria, Biodegradation, Bio-methanation, Coal, Fungi, Humic acid-like substances, Rehabilitation

## Abstract

Coal mining and coal combustion for energy generation will continue in the medium term and remain a primary source of pollutants. Its complex structure renders coal a recalcitrant material and relatively few bacteria and fungi can thus degrade this carbonaceous substrate. In this review, we assess research progress on the biological degradation and solubilisation of coal, waste coal, discard and gangue from 2014 to 2024, the period following the publication of our 2013 critical appraisal of this topic. We focus on the continued need for studies on coal biodegradation and bio-solubilization. We explore and, where appropriate, evaluate some of the more important recent advances in coal bio-solubilization research to illustrate progress in this field. Of particular significance are the ever-increasing number of bacterial and fungal biocatalysts identified as possessing coal degrading potential, the role of microbial consortia in this process, the aerobic and anaerobic mechanisms of coal utilisation, and progress in elucidating the underlying molecular and biochemical events involved. Also reviewed are advances in the application of industrial products derived from coal, including biomethane, coal-bed methane, and humic substances, and the use of waste and discard coal-derived humics as technosols for soil restoration and the commercial-scale rehabilitation of coal mining-affected land. It is concluded that an understanding of the mechanisms underpinning coal biodegradation is critical in combating many of the detrimental impacts of mined coal, exposed coal seams and stockpiled coal mine waste and that the outputs from these studies must be incorporated into the development of diversified production technologies and strategies for both socio-economic and ecological gain.

## Introduction

In an earlier review on the biological degradation and solubilisation of coal we reported on and discussed in detail the fungal and bacterial catalysis of the process and the various biochemical mechanisms thought to be involved (Sekhohola et al. 2013). Furthermore, we presented a conceptual model that sought to describe phyto-bioconversion of low rank coal by illustrating the potential interactive pathways between ligninolytic microorganisms and root exudate of plants growing on soil rich in this carbonaceous material sometimes, pollutant. It was concluded that coal is depolymerised in a complex process that is driven by extracellular microbial enzymes with solubilisation facilitated by alkaline substances and surfactants. Also highlighted was progress at the time on fundamental research into the biochemical processes underpinning coal biodegradation (Sekhohola et al. 2013). Aerobic and anaerobic degradation can occur and both result in the accumulation of microbial biomass, insoluble organics and humin, and release of carbon dioxide (CO_2_), methane (CH_4_), and water. In short, competent microbes mineralize coal to access carbon and to obtain nutrients to sustain their growth which enhances accessibility of the substrate for further microbial processing and utilization. Initiation of the process requires contact between the substrate and the biocatalyst(s), alterations in microbial gene expression, and changes in the formation and function of gene products. Subsequent and sequential depolymerisation and hydrolysis of the macromolecule to simpler forms that serve as an energy source for microbial growth occur coincident with the appearance of novel metabolites and non-metabolizable species. The latter appear to accumulate as a suite of substances i.e., humic acid, fulvic acid, and humin, collectively referred to as humic substances hereafter (Sekhohola et al. 2013). Bio-methanation, the conversion of coal to the fuel gas CH_4_, by anaerobic microbial activity, is important for energy production. It involves a consortium of microorganisms, including methanogenic archaea, which can be optimised for maximum CH_4_ yield (Tiago et al. 2025). Thus, both aerobic and anaerobic biosolubilization bring about the degradation of coal.

Earlier research (prior to 2013) concentrated on laboratory-scale screening of microorganisms for coal biotransformation potential, the development of methodologies to probe the various mechanisms thought to be involved in bioconversion, and identification of products at each stage of coal bioprocessing. A gap identified at the time was the lack of and limited information about upscaling of coal biodegradation processes to industrial or commercial scale. In particular, the emergence and optimisation of rehabilitation technologies for soil restoration of coal mining-disturbed land. Since that time, we, and others have elaborated strategies for rehabilitation of land disturbed by coal mining (Cowan et al. 2016; Widhayasa and Susanto 2019; Xu et al. 2023) and similar anthropogenic actions (Coban et al. 2022). Furthermore, continued microbial (Li et al. 2023b; Xia et al. 2024), biochemical (Ghani et al. 2021; Li et al. 2023a; Niu et al. 2023) and molecular (Kwaitos et al. 2018, 2020) studies have resulted in a much deeper understanding of the coal biodegradation process and beneficiation technologies that have been elaborated on at least two fronts (Sekhohola-Dlamini et al. 2022; Akimbekov et al. 2022).

In the review by Akimbekov and co-workers (2022), the focus is on coal ecosystem-derived microorganisms with potential, either intrinsic or acquired, to impact coal biotechnology by providing novel products and processes for development of cleaner coal technologies including land restoration and rehabilitation. In the other, a perspective by Sekhohola-Dlamini et al. (2022), a novel bioprocess for the sustainable rehabilitation of mining-disturbed and degraded land was elaborated to provide the underpinning details of this biotechnological strategy which has at its core, the in situ bioconversion of coal discard into humic substances capable of regenerating soil fertility and supporting plant growth. Indeed, a recent review by Lu et al. (2024) has expanded on the earlier hypothesis of Sekhohola-Dlamini et al. (2022) by focussing attention on the role of bacteria and fungi as key biocatalysts in soil reconstruction.

The present review discusses advances in  the study of coal biodegradation from 2014 to 2024, with a focus on microbial mechanisms and associated application. Of particular significance is the ever-increasing number of biocatalysts capable of degrading coal and the emerging role of microbial consortia in this process. We discuss at length recent developments in coal biosolubilization technology in terms of bio-methanation and coal-bed methane (CBM), humic substance production, the use of coal as a soil amendment, and in myco-phyto-bioremediation strategies for the rehabilitation of coal mining-affected land.

## Importance of studies on coal biodegradation/biosolubilization

Perhaps the most important reason underpinning studies on the biological breakdown of coal and its liquefaction is to ensure a just transition from coal mining and combustion for energy generation to responsible beneficiation and sustainable land (soil) restoration to allow for its productive use following mine closure. Historically, coal mining has been a major contributor to economic growth in many countries around the world. Coal was once an abundant and desired fuel and was sourced for use in heating and later, for bulk electricity generation. Many countries invested in mining for the export of coal as a commodity product. Today, coal mining is still practiced in parts of the world and is a driver of many national and regional economies that are heavily invested in and reliant on this resource for energy. So, coal and more recently oil and gas have fuelled the economic engines of many of today’s most prosperous nations but not without consequence including production of pollutants and copious amounts of solid waste (also referred to as gangue). The waste, discarded or stockpiled in the form of massive dumps and tailings dams, continues to be a source of coal-derived pollutants. Following precipitation events, the products of coal oxidation accumulate in the soil and ground water and also are released to the atmosphere as an increase in particulates. It has also been suggested that coal mining increases the risk of antibiotic resistance gene (ARG) proliferation. However, an analysis of coal mining-derived acid mine drainage (AMD)-contaminated soil showed lower relative abundance of ARG but increased heavy metal(loid) resistance genes (MRG) and levels of mobile genetic elements (Huang et al. 2023). These finding seem to support the idea that the co-occurrence of MRGs and ARGs arises because of co-resistance, cross-resistance, and coregulation as proposed by Li et al. (2017) and Pal et al. (2017). Further examples of the extensive and devastating historical impacts of coal mining waste on the environment include the cases of the Canadian Rocky Mountain and the Appalachian Mountain coal fields (Cooke et al. 2024; Zipper and Skousen 2021). Lasting impacts of mining and waste disposal in these regions include deleterious effects on soils, plant communities, wildlife, and water sources and effort to rehabilitate or restore the affected land and water sources, at a cost of nearly US$ 7.2 billion, did not yield the expected outcomes (Lima et al. 2016). These authors concluded that large scale ecosystem restoration *in sensu stricto* is unlikely to be successful (Lima et al. 2016). This contrasts with reports of the successful commercial scale rehabilitation of land disturbed by coal mining and of waste coal dumps using a myco-phyto bioremediation strategy based on fungal biosolubilization of the carbonaceous pollutant (Cowan et al. 2016; Sekhohola and Cowan 2017). This situation notwithstanding, coal mining world-wide continues to produce large volumes of solid waste or coal discard, which is a significant source of pollutants that exacerbate degradation of arable land and render it infertile and unproductive (Howaniec et al. 2018; Pierwoła et al. 2018; Pactwa et al. 2020; Adhikari and Mal 2021; N´adudvari et al. 2021).

In view of the risks attached to stockpiled waste, it is clear that there is a need to expand the management of coal discard beyond disposal and eventual rehabilitation. Coal disposal and management methods ought to evolve towards more sustainable approaches that reduce the associated risks and liabilities, and incorporate optimised and efficient utilisation of all mined coals. Waste coal is an untapped resource with potential to be a raw material for bioprocessing. There are two areas of opportunity that should be explored further; 1) chemical and biological degradation and solubilization of coal and transformation into humic acid-like organic material for use as a soil amendment in carbon-depleted soils, and 2) conversion of coal in large-scale bioreactors to produce a number of very useful and desirable industrial products including, but not limited to humic acids, volatile fatty acids, ligninolytic enzymes, and methane-rich biogas.

Today, many coal mining regions are subject to strict regulatory authorization. This authorization requires the rehabilitation and/or reclamation of land disturbed by mining, and many companies are mandated to have sound rehabilitation protocols in place before, during, and after mining has stopped and prior to mine closure (Boulot and Collins 2023). There are, however, major limitations to the reuse of coal as a substrate for beneficiation or as a targeted pollutant in the development of rehabilitation strategies, particularly where dumped waste is of unknown calorific value and remains uncharacterised in terms of chemical content and composition (Onifade and Genc 2018). Thus, extensive characterisation of stored coal wastes is seemingly necessary to determine potential risks to support the development of appropriate and effective rehabilitation strategies (Williams et al. 2019). On the other hand, characterisation of waste coal does allow for this material to be repurposed and recycled for downstream beneficiation. Consumption of waste through the emergence of parallel industries may significantly reduce the quantity of discard destined for waste dumps and tailings dams and help in mitigating the overall impact of coal mining. For example, in South Africa, thorough characterisation of waste coal using particle size analysis, sink-float studies, and static tests resulted in close to 70% of the coal discard being pooled for future use as it was of sufficient quality for energy generation in conventional power plants, while the rest it was suggested, could be used to fabricate technosols or other aggregate materials (Filho et al. 2020). Taha et al. (2023) also recount an integrated and circular approach explored in Morocco to recover and recycle dumped anthracite coal for energy recovery, brick and concrete production, and manufacture cleaner-burning coal briquettes.

The question, of whether industrial bioprocessing of coal is feasible for the efficient use of all coal forms that would otherwise be discarded as waste, remains. So far, small-scale research has indicated that biological technologies, operated under conditions of ambient temperature and pressure are potentially an effective alternative approach to thermochemical conversion technologies used for liquefaction and gasification of coal. The latter require energy-intensive operations and costly downstream separation (reviewed by Li et al. 2023d). Furthermore, microorganisms continue to be isolated from mined soil and coal environments, enriched and optimized for coal biodegradation, making microbial mediated coal degradation a viable beneficiation strategy for alternative fuels and non-fuel organics. These advances should ultimately provide an in-depth understanding of the process dynamics for use in developing commercial technologies based on coal bioconversion and transformation to high-quality liquid or gaseous fuels, liquid chemicals, solid organic-rich substrates, and other high-value products.

## Coal biodegradation and biosolubilization: aerobic and anaerobic mechanisms

The structural organic fraction of coal consists of a complex mixture of aliphatic and aromatic hydrocarbons together with oxygen-, nitrogen-, and sulfur-containing heterocyclic compounds. This structural chemistry is complex, heterogeneous, and perhaps not surprisingly, all coal degrading microorganisms possess an arsenal of mechanisms that, in addition to peroxidation activity, cleave the various molecular substructures into smaller fractions. These mechanisms include the production of **a**lkaline substances, **b**iocatalytic oxidation, **c**helation, **d**etergent and surfactant production, and increased activity of extracellular enzymes such as **e**sterases which led to the proposed ABCDE model of coal biodegradation (Klein et al. 2001). For the most part and as discussed below, the predominant mechanisms operative in the biological breakdown of coal appear to include all, some, or combinations of these processes (Sudheer et al. 2016; Akimbekov et al. 2020a; 2020b, 2021a, b).

Convention seems to indicate, at least from legacy studies, that pretreatment of coal, lignite, and coal discard is necessary to facilitate biodegradation. Thus, many early and contemporary studies on the biotransformation of coal and waste coal considered it essential to pretreat the substrate material with concentrated acid, alkali, or other oxidizing agents. With the discovery of many microbes with coal degrading capability, pretreatment of the substrate with either acid or base has become less common and for the most part has been overlooked or omitted entirely. Even so, treatment of lignite using increasing concentrations of HNO_3_ (i.e., 2N, 5N, and 8N) showed that treatment at 5N was most effective for coal biosolubilization by *Streptomyces fulvissimus* K59 in a liquid culture (Sobolczyk-Bednareka et al. 2021). This again raises an interesting question that was in part addressed by Olawale et al. (2020). These authors argued that along with the addition of nitrile groups to aromatic substructures in coals and lignites, nitrosation reaction products have also been detected in HNO_3_-treated coals as was formation of secondary reaction products of the initially formed oximes (Thorn and Cox 2016). Furthermore, and as shown in detailed NMR studies (Thorn and Cox 2016), the final oxidation step of phenol with nitrous acid regenerates nitrous acid, which will likely continue in perpetuity and react as a nitrosating agent (i.e., introduces nitroso (–N = O) groups) leading ultimately to structural changes in molecular integrity, substrate dissolution and negate or diminish the bulk contribution apportioned to the various biological catalysts being screened. Other oxidising agents that have been used include solutions of potassium permanganate (KMnO_4_) and hydrogen peroxide (H_2_O_2_). Indeed, the latter has been successfully used to prepare fulvic acids (FA) from lignite using microwave irradiation with CuO as catalyst (Gong et al. 2022). Products were structurally characterised using elemental analysis, infra-red (IR) spectroscopy and ^13^C nuclear magnetic resonance (NMR) spectroscopy and shown to possess the chemical formulae C_44_H_30_O_25_N and C_53_H_35_O_31_N. The relative abundance of the major functional groups was carboxyl > phenolic hydroxyls > alcohol hydroxyls > carbonyl.

Biosolubilization of coal is a process that can potentially transform all types of coal, coal discard, coal fines, and waste coal into products that may be of commercial value, have use in cleaner energy generation, and/or act as substrates in bioprocesses developed to synthesise or refine complex aromatics (Ghani et al. 2015). Both aerobic and anaerobic degradation can lead to biosolubilization and for this to occur, contact between the various biocatalysts and the substrate is essential and, the greater the contact or surface interaction the greater the probability of bioconversion. Indeed, for many microorganisms the formation of aggregated layers, filaments, and the production of exudates or extra-polymeric substances (EPS) facilitate biofilm formation and are part of the mechanism of attachment that facilitates biochemical interaction between biocatalyst and substrate (Sekhohola et al. 2014; Olawale et al. 2020).

Both Gram-negative and Gram-positive bacteria have hair-like structures on the surface of the cell called pili or fimbriae (Proft and Baker 2009). Most commonly, these structures are involved in the adherence of cells to a variety of different surfaces including other bacterial cells. Composed of pilin proteins, pili facilitate attachment to surfaces and contribute to the formation of biofilms and colonisation (Hu et al. 2016). For example, in *Pseudomonas aeruginosa,* type IV pili act as mechanosensory organs to detect surface contact through mechanical activity. This sensing appears to trigger a chemosensory signalling cascade that regulates virulence through force-induced conformational changes that induce a cascade of molecular events including c-di-GMP signalling and biofilm formation (Persat et al. 2015; Webster et al. 2022). Bacterial filaments also participate in colonisation. Formation of these elongated, thread-like structures is due to incomplete cell separation post binary fission and a survival strategy to counter stressful growth conditions by promoting surface colonisation (Tran et al. 2022). This is particularly true for environments that experience or are exposed to extreme conditions such as the unprotected surface of coals and similar substrates. Detailed studies on mechanisms used by human bacterial colonizers to mitigate the effects of physical stress include production of multilayered aggregates or biofilms and tacky matrices comprising lipid, protein and sugar polymers that together protect the microbes from removal (Otto 2014; Hallinen et al. 2025). Similar mechanisms undoubtedly operate in the colonization of coal by microbes to facilitate its biodegradation. A study using bespoke polished coal disks derived clear evidence supporting the involvement of bacterial biofilm production and changes in community species structure and composition during colonization (Vick et al. 2016). And the influence of surface physico-chemistry on cell attachment to coal, using *Pseudomonas fluorescens* Pf-5 a well-known coal-oxidizing bacterium as a model has been described (Hazrin-Chong et al. 2021). For other microbes, including fungi and yeasts, interaction may include an increase in hyphal peroxisome biogenesis together with elevated bioconversion activity in aerobic environs and in anoxic zones, and where coals are more oxidized than bituminous-type coals, elevated methanogenic potential is the most observed outcome.

Peroxisomes play an important role in substrate channeling (Song et al. 2024). Examples include the formation of acetyl-CoA from β-oxidation of fatty acids and detoxification of substrate xenobiotics for supply of carbon to heterologous pathways. Closer interactions between substrate and biocatalyst results in liberation of larger quantities of small organics that act as precursors for further biodegradation. Interestingly, the distribution of methanotrophic bacteria in lignite, explored using methanotroph-specific lipid biomarkers, revealed that these are associated with elevated bacteriohopanepolyols (Pytlak et al. 2021). And a geochemical study of Leonardite-like weathered coal from the Emalahleni coal fields in South Africa showed that this material was enriched with fatty acids (16:0 and 18:0) and chemical signatures including homohopanes (van Breugel et al. 2019). The latter occur commonly in sedimentary rock and in various coals and lignites and are likely a residue of prokaryote activity from both fossil and living bacteria, such as methanotrophs. Thus, it is perhaps not surprising that several species of bacteria, particularly those isolated from coal-bed methane (CBM) seams, coal discard dumps, and soils polluted with coal hydrocarbon, are able to colonize and oxidatively degrade coal (Hazrin-Chong and Manefield 2012; Hazrin-Chong et al. 2014; Vick et al. 2016; Olawale et al. 2020).

**Fig. 1:**
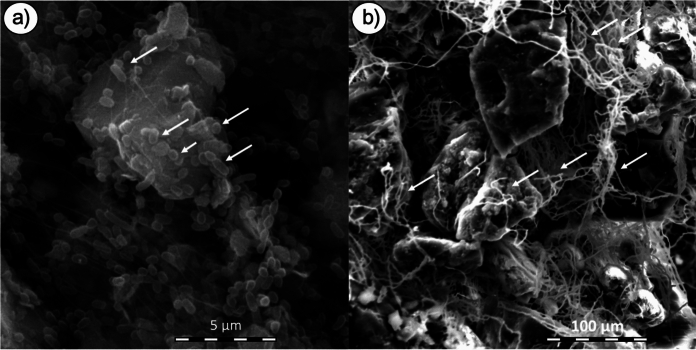
Microbial colonization of coal particles. Scanning electron micrographs of **a** colonization of the coal surface by *Bacillus* strain ECCN 41b (phenotypically idential to *Priestia megaterium*); and, **b** colonization and hyphal engulment of coal particles by the fungus *Neosartorya fischeri* strain ECCN 84 cultured in liquid media containing basal salts medium and coal as the sole carbon source. Micrographs are courtesy of Ms Lwazi Madikiza **a** and, Dr Lerato Sekhohola-Dlamini **b**

Figure 1 illustrates what is typically observed during colonization of coal particles by bacteria (Fig. 1a) and fungi (Fig. 1b). In the latter, ultrastructural investigation revealed an increase in the number of peroxisomes along the periphery of hyphal cells and closest to the coal particle surface (Sekhohola et al. 2014). Peroxisomes are the organelles which are rich in enzymes that catalyze diverse oxidative and metabolic reactions including those involved in detoxification, lipid metabolism, antibiotic production, and reactive oxygen species (ROS) metabolism amongst others (van der Klei et al. 2002; Meijer et al. 2010; Steinber 2016). While unconfirmed, the implications are that peroxisome biogenesis likely coincides with the presence of xenobiotics and the onset of signalling which triggers oxidative enzyme synthesis and metabolism.

In aerobic environments, degradation of coal continues to be considered as a series of oxidation reactions catalyzed by oxidizing agents and oxidative enzymes culminating in the release of volatile products including CH_4_ and CO_2_ (Table 1). Bacterial degradation and utilization while impacted by the environment including precipitation, temperature, and pH, is reliant on the type of coal and suite of microorganisms that are present (Titilawo et al. 2020; Yu et al. 2020) and like fungi, the mechanisms include solubilization, depolymerization and degradative assimilation (Sekhohola et al. 2013; Akimbekov et al. 2022; Sekhohola-Dlamini et al. 2022). Generally, biosolubilization is believed to be by anyone or a combination of alkaline substances, chelators and/or surfactants, whereas depolymerization and liquefication make use of either O_2_ or H_2_O_2_, or H_2_O, as reactants, involves oxidoreductases such as peroxidases and laccases, andor hydrolases respectively. Nitrification, which is the biocatalyzed sequential conversion of ammonia to nitrite and nitrate, is also thought to participate in the coal biodegradation process (Gutierrez-Zamora et al. 2015). The latter was in part confirmed by addition of the nitriﬁcation inhibitor allylthiourea (ATU) to active sub-cultures of a coal degrading microbial consortium isolated from cored coal. Products formed under aerobic conditions include biomass and metabolites of fungal and bacterial origin e.g., phthalic acid, di(2-acethylphenyl) ester, coal-derived hydrocarbons, humic and fulvic acid, humin, NO_3_, CH_4_, CO_2_, and H_2_O amongst others. In contrast, under anaerobic conditions biodegradation of coal involves various functional genes that encode enzymes specific to the anaerobic process. Under these conditions, biodegradation is by hydrolytic and fermentative pathways in which a consortium of microorganisms converts coal-derived compounds into organic acids, alcohols, and the respiratory products CH_4_ and CO_2_. Thus, the anaerobic process yields microbial biomass, the gases CH_4_, CO_2_, H_2_, NH_3_ and H_2_S, soluble ammonium, and insoluble humics and humin as products. Additionally, the presence of specific microbial (i.e., bacterial and archaeal) metabolites might also be expected. A comparative assessment of the aerobic and anaerobic processes is summarized in Table 1.

**Table 1:** Comparison between aerobic and anaerobic biodegradation pathways based on similarities and differences, and advantages and disadvantages

Characteristic	Aerobic biodegradation	Anaerobic biodegradation
Microbial communities involved	Aerobic bacteria, actinomycetes, fungi	Anaerobic bacteria (fermenters, acetogens, sulfate-reducers) and Archaea (methanogens)
Primary electron acceptor	Oxygen (O₂)	Alternative electron acceptors such as Nitrate (NO₃⁻), Sulfate (SO₄^2^⁻), Carbon dioxide (CO₂), Ferric iron (Fe^3^⁺), or protons (H⁺)
Mechanism of organic matter degradation	Complete mineralization of organic compounds to CO₂, some CH_4_ and H₂O via aerobic respiration	Stepwise breakdown: Hydrolysis → Acidogenesis → Acetogenesis → Methanogenesis (multi-step and syntrophic interactions)
End products	CO₂, (CH_4_), H₂O, Biomass	CH₄, CO₂, H₂, Volatile fatty acids, Hydrogen sulfide (H₂S), Biomass
Energy yield (ATP generation)	High energy yield (~ 38 ATP/glucose)	Low energy yield (~ 2–4 ATP/glucose), dependent on pathway and final electron acceptor
Degradation rate	Generally faster due to higher energy gain and more efficient metabolism	Generally slower due to limited energy availability and multi-step process
Environmental conditions required	Requires dissolved oxygen (DO), optimal DO levels usually > 2 mg/L	Strict anaerobic conditions (DO < 0.1 mg/L), sensitive to oxygen exposure
Operational complexity	Requires aeration systems (energy-intensive)	Requires sealed, oxygen-free reactors, often with gas handling systems
Advantages	Rapid biodegradation, complete mineralization, fewer intermediates, suitable for surface environments	Energy recovery in the form of methane; suitable for deep subsurface or oxygen-limited environments; lower sludge production; less requirement for N and P; source of pathogenic organisms; less spatial requirements; cost-effective; high removal efficiency
Disadvantages	High operational cost due to aeration, not suitable for deep/anaerobic zones, potential for incomplete degradation under low oxygen	Slow degradation rate, sensitive to pH, temperature, and toxic compounds, risk of intermediates accumulating (e.g., VFAs)
Applications	Wastewater treatment (activated sludge), soil bioremediation, composting	Anaerobic digestion, biogas production, landfill treatment, subsurface bioremediation, coal or oil degradation in deep formations
Typical carbon conversion efficiency	High conversion to CO₂ with biomass production	Conversion to CH₄ and CO₂ with some biomass; often more carbon ends up as biogas

**Fig. 2:**
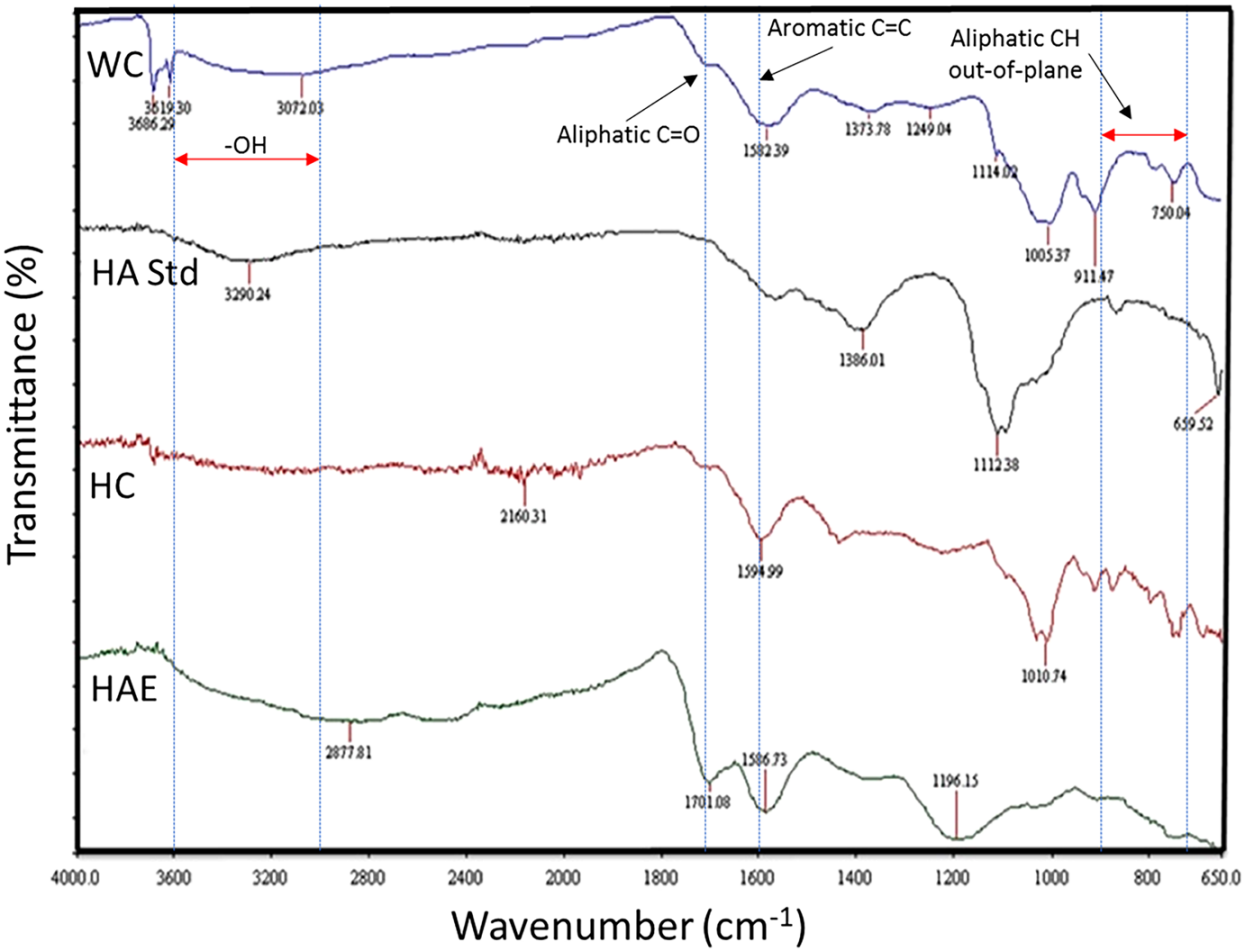
Fourier transform infrared spectra of waste coal (WC), bituminous hard coal (HC), commercial humic acid (HA), and WC humic acid extract (HAE). Bands from 3600-2000 cm^-1^ show the presence of carbonyl, hydroxyl, carboxyl, and amide groups. These groups are absent in the bituminous HC sample but present in WC, confirming the latter as more oxidised and weathered. FTIR spectra from samples of commercial HA and the coal-derived HAE are similar in terms of functional groups, although signal intensity differs

Whether the degradation of coal is by aerobic or anaerobic bioconversion, Fourier transform infrared (FTIR) spectroscopy is one laboratory method routinely used to analyze the products and residue to confirm oxidative depolymerization and an increase in oxygen functional groups following inoculation and incubation with various microbes. Results are typically compared to spectra generated from substrate coal and from known standards. By way of example, the FTIR spectra of alkaline-extracted humic acid (HAE) from waste coal (WC), bituminous hard coal (HC) and authentic humic acid (HA, Sigma Chemical Company) are shown in Fig. 2. To interpret this spectral data, band assignments follow the method of He et al. (2016) concerning published data reported for region-specific bituminous coals (Okolo et al. 2015) and the physicochemical characteristics of coal-derived alkali-extracted HA (Wang et al. 2017a).

In addition to the above, greater effort is needed to isolate and physicochemically characterize the water-soluble and insoluble products of coal biodegradation to establish the identity of specific chemical signatures. These may be either coal or biocatalyst specific. Methods should include excitation-emission matrix spectroscopy (EEMS) for qualitative estimation of the released organics and/or pyrolysis-gas chromatography-mass spectrometry (pyr-GC-MS) of solid or insoluble residue, and combined gas chromatography-mass spectrometry (GC-MS). The latter approach was elegantly used by Haider et al. (2013) to confirm the formation of single-ring aromatics, polyaromatic hydrocarbons (PAH), aromatic nitrogen compounds and aliphatics as products of coal biodegradation by a novel isolate of the fungus, *Penicillium chrysogenum*. And more recently, Xia et al. (2024) showed using EEMS (more specifically 3D-EEM) and GC-MS that coal hydrolysis products were predominantly aromatic compounds and organic acids and that the responsible bacterial biocatalysts, *Bacillus polymorpha* and *Bacillus sphaericus*, had high hydrolytic activity ideal for pretreatment of coal in its anaerobic processing to CH_4_.

## Aerobic biodegradation of coal

Most studies on the aerobic biodegradation of coal, low-rank coal and waste coal have utilized a traditional microbiological approach (Sekhohola et al. 2013; Akimbekov et al. 2022). Various fungi or bacteria sourced and isolated from environmental samples have been screened for biodegradation potential and any that respond positively are enriched by cultivation in a minimal medium with coal as the sole source of carbon (see for example, Olawale et al. 2020; Khan et al. 2024a). To date, this approach has been only marginally successful with a laccase (LAC, E.C. 1.10.3.2) and an esterase being the only candidates identified and functionally characterized (Kwiatos et al. 2018; Yang et al. 2018a, b).

As the combustion of coal for energy is now actively being discouraged, the bioconversion of coal to yield clean and cost-effective products has become more intense. One potential coal processing technique currently being explored is the application of microbes and their derivatives to modify the surface structure of coal for downstream processing. And a possible indirect outcome may of course be a better understanding of the mechanism underpinning coal biodegradation. For example, in one study the white-rot fungus *Hypocrea lixii*, including its spores, hyphae, EPS, and residual culture solution, and in an aerobic environment, effectively improved the surface properties of treated coal (He et al. 2023). Spectroscopy (i.e., FTIR) revealed that the EPS fraction caused an increase in carboxyl, hydroxyl, and amidogen functional groups indicative of surface modification. Most effective was EPS > culture solution > hyphae > spores. In a related study, the degradation of carbonaceous matter in refractory gold ores was tested by treatment with a cell-free spent medium (CFSM) containing lignin degrading enzymes produced from the fungus *Phanerochaete chrysosporium* (Konadu et al. 2023). Raman and 3D-fluorescence analysis confirmed that the fungal-derived lignin-degrading enzyme treatment improved ore extraction by reducing more readily the non-graphitic organic carbon content. Additional studies targeting fungi as the biocatalyst have identified *Penicillium aculeatum* 13–2-1 from the rhizosphere of *Zelkova serrata* as a degrader of weathered coal (Ren et al. 2023), shown that lignite biodegradation by *Fusarium sp*. NF01 changes the surface structure and increases the free radical content of residual lignite (Niu et al. 2022), and identified the fungal isolate NF-2 as *Aspergillus flavus*-like based on its ability to convert coal to humic substances with a humification index value (A4/A6) of 6.18 compared to the standard A4/A6 value of 4.27 for commercially available humic acid (Rehman et al. 2022). Together, results from these recent studies confirm the versatility of fungi as biocatalysts of choice for aerobic coal biodegradation with the formation of humic substances as a major outcome. Similarly, small-scale pot and in situ commercial-scale trials demonstrated fungal-induced oxidative conversion of low-grade coal discard to a humic substance-enriched soil-like material (Cowan et al. 2016; Sekhohola and Cowan 2017). Unfortunately, the only confirmed mechanism for the biological degradation of coal by fungi remains the biosolubilization of brown coal by a novel recombinant *Fusarium oxysporum* LAC (Kwaitos et al. 2018) and the oxidative depolymerization of lignite by an esterase from *Penicillium decumbens* P6 (Yang et al. 2018a, b).

For bacteria, the situation is more complex as there has been very little advance in the details that underpin the biochemical mechanisms responsible for biosolubilization and transformation of coal. Nevertheless, the past decade has seen an increase in efforts to screen and use various bacteria to biodegrade low-rank coals (Olawale et al. 2020; Hazrin-Chong et al. 2024; Levania et al. 2014; Valero et al. 2014; David et al. 2017; Akimbekov et al. 2021b, a; 2020a) and some of the isolated coal-degrading bacteria appear to display traits typical of plant growth-promoting rhizobacteria (Titilawo et al. 2020). Furthermore, the idea of using a ‘bioaugmentation’ approach has been proposed (Wang et al. 2016). These authors showed that ‘natural’ coal is a “seed bank” of bacterial strains that are active lignin degraders. And as so eloquently described by Kozhakhmetova et al. (2024), bioaugmentation or the addition of pure microbial strains to native populations within the target substrate successfully enhance liquefaction of coal, and stimulate biogenic CH_4_ and biogas production. In their study, activated sludge (AS) was used as a supplement to augment and enhance bioconversion of coal into smaller organic complexes with properties that resemble humic substances.

It seems therefore that the biological degradation of coal catalysed by bacteria and/or fungi proceeds via a liquefaction step to an end-product which, depending on prevailing conditions will comprise microbial biomass, the biogases CO_2_ and/or CH_4_, and the much less volatile humic substances. Biological gases are desired products in anaerobic reactors and CBM seams whereas humic-rich material appears desirable for aerobic processing to higher value products. And it remains distinctly feasible that the biochemistry underpinning Klein’s ABCDE model of coal breakdown proposed in 2001 (Klein et al. 2001) contributes to both. These, and other more recent advances in the oxidative mechanisms of coal biodegradation and biosolubilization are summarised below.

## Biodegradation by alkaline substances, chelating agents, and detergents

Many bacteria capable of producing alkaline substances, chelating agents and detergents or surfactants play a key role in the biodegradation of complex substrates like coal. By exploiting one or a combination of these mechanisms the oxidation of the substrate is enhanced. Alkaline substances (e.g., ammonia, amines, peptides, etc.) and chelators (e.g., oxalic acid, salicylic acid, and triethylamine) are small molecules produced by microorganisms that solubilize (liquefy) coal. Chelating agents secreted by microorganisms can react with metal ions including Ca^2+^, Fe^2+^, and Mg^2+^ in coal to depolymerize its molecular structure, resulting in the generation of smaller water-soluble molecules. Bacterial surfactants, also known as biosurfactants, are small detergent-like amphipathic molecules produced by bacteria that reduce surface and interfacial tension to promote pollutant bioavailability. Thus, surfactants enhance coal solubilization by promoting absorption of enzymes onto the surface of the coal substrate and by reducing surface tension. In addition, surfactants may alter the binding of certain enzymes leading to higher coal biodegradation rates. For example, in recent studies the soil-isolated bacteria, *Cupriavidus necator* S2A2, *Sphingopyxis ginsengisoli* S2B14, and *Sphingomonas sp*. S2B18 grown in LB medium containing 1% coal were found to solubilize untreated low-rank coal which was confirmed by the release of solubilized coal products detected at OD_450_ (Baylon et al. 2017). In the absence of any detectable enzyme activity, it was concluded that the observed biosolubilization was due to the excretion of bacterial-derived alkaline substances. And a metabolomics study identified the breakdown products generated by cultures of the flavobacterium *Planomicrobium huatugouensis* supplied with Dananhu low-rank coal as the carbon source (Yang et al. 2023). Results revealed that 43 metabolic products were upregulated and included the alkaline substances ammonia, tyramine, N-acetyl cadaverine, L-carnitine, betaine, and citrate. Evidence was also provided to suggest that these metabolites acted on ester, ether, and metal linkages to liquefy substrate coal which yielded alcohols, aldehydes, and ketones (Yang et al. 2023). And the biodegradation of tar-rich coal could be achieved by *Bacillus licheniformis* using either biological (Rh), non-ionic (Triton X-100), anionic (LAS) or cationic surfactants (DTAB). Rhamnolipid (Rh), Triton X-100, and LAS promoted bacterial growth by increasing the cell surface hydrophobicity which enhanced the biodegradation of aromatic and aliphatic structures of oxidized coal (Shen et al. 2023).

## Enzyme-catalyzed biodegradation of coal

Several enzymes have the potential to degrade coal and release bioavailable substances for further microbial assimilation or for use as co-substrate polymerization reactions and the formation of soil organic matter (Sekhohola-Dlamini et al. 2022). Low-rank coal and lignite or brown coal have been pre-treated with enzymes prior to utilization for energy generation and materials synthesis. Treatment of lignite is usually by oxidoreductase-type enzymes including LAC, horseradish peroxidase (HRP), alcohol dehydrogenase (ADH), and has revealed that LAC treatment leads to formation of larger coal aggregates likely due to enhanced crosslinking. Indeed, 876 bacterial strains from 27 genera belonging to the Actinobacteria, Firmicutes, and Proteobacteria, showed that 612 were positive for lignin degradation (Wang et al. 2016). Dominant lignin-degrading strains included *Thauera*, *Arthrobacter*, and *Rhizobium* and genes encoding LAC- and LAC-like oxidases were found in three genera including a *Massila* spp. In contrast, both HRP and ADH treatment either lowered the aliphatic or increased the aromatic hydrocarbon content, particularly in lignite pre-treated with ionic liquids. And treatment with either ADH or LAC increased the relative abundance of hydrogen bonds which likely improved the overall stability of the molecular structure of this biopolymeric complex (Zhao and Baker 2022). Pretreatment may also be useful for processes such as the grafting of MnO_2_ particles onto low-rank coal to produce adsorbents for water purification (Abbas et al. 2024) and in the modification of coal fly ash (CFA). For example, properties such as porosity, surface area morphology and chemical composition seem to improve the efficiency of CFA material for wastewater treatment or for its converted to a geopolymer for use as an adsorbent and photocatalyst (Singh et al. 2022a). Some of the most effective enzymes considered to be involved in the microbial solubilisation of coal are discussed in detail below.

*Lignin peroxidases* – These enzymes are topical owing to their high redox potential. Lignin peroxidase (LiP, E.C. 1.11.1.14) produces hydroxyl radicals which directly react with aromatic ring structures leading to C–C bond breakage and the formation of long-chain alkanes (Liu et al. 2021; Teo et al. 2023). Lignin peroxidases were also reported to destroy part of the C–C bond of low-rank Heishan coal and opened the aromatic ring (Shi et al. 2024). That larger macromolecules could be degraded into small aliphatic and aromatic compounds by Li-P secreted by bacteria had previously been observed (Okolo et al. 2015). The analysis of lignite and its liquefaction products using ^13^C NMR showed that a decline in aromatic carbon was associated with an increase in phenol aromatic ether carbon and oxygen-linked fatty carbon, which suggested that LiP had catalysed ring-opening, oxidation, and other reactions during lignite liquefaction (Shi and Wang 2019).

*Laccases* – These are Cu^2+^-containing polyphenol oxidases produced by almost all lignin-degrading fungi and molds. Laccases (LAC, E.C. 1.10.3.2) have a wide range of substrates and can catalyze the oxidation of various substrates such as monophenols, polyphenols, aromatic amines, and non-phenolic compounds. A role for LAC in the biodegradation of coal dates to the 1980s, and first evidence for a fungal LAC activity was reported for the fungus *Neosartorya fischeri* strain ECCN 84 in 2014 (Sekhohola et al. 2014). It was shown earlier that this strain was capable of liquefying hard coal (Igbinigie et al. 2008) and that it produced an extracellular oxidative LAC that mineralized petroleum asphaltenes (Uribe-Alvarez et al. 2011). Thus, it was perhaps expected that culture filtrates of this strain exposed to coal would test positive for LAC using the indicator dye 2.6-dimethoxyphenol, 2.2′[azino-bis-(3-ethylbonzthiazoline-6-sulphonic acid) diammonium salt] (ABTS). As far as the authors can establish, LAC remains the only known enzyme that has been identified and functionally characterized from a coal-degrading microorganism. Kwiatos et al. (2018) were first to report that a *Fusarium oxysporum* LAC could be expressed in the yeast *Pichia pastoris*, which was assessed as an additional agent for coal degradation. The expressed LAC contributed to humic and fulvic acid release due to introduction of oxygen into the coal structure (Kwiatos et al. 2020). Interestingly, microbes with the potential to degrade polyethylene were isolated and used to demonstrate the close association between the biodegrading capability of the isolates *Pseudomonas aeruginosa* O1-P and *Bacillus cereus* O2-B and LAC (Farveen et al. 2023). Wang et al. (2016) showed that treatment with LAC led to formation of larger cross-linked coal aggregates with higher relative abundance of hydrogen bonds that improved the overall stability of the molecular structure of this biopolymeric complex (Zhao and Baker 2022) Further, different bacteria with lignin degrading potential have also been isolated from coal and shown to possess genes that encode LAC- or LAC-like multicopper oxidases which are crucial for coal degradation (Zhao and Baker 2022). These genes were identified in three genera: *Aerococcus*, *Shinella*, and *Massilia*. However, a comparison of the phylogenetic tree of LAC genes revealed that strains possessing similar LAC genes can be quite different based on 16S rRNA gene sequence analysis. This observation suggests that there might be significant horizontal gene transfer of LAC genes between strains isolated from coal (Wang et al. 2016).

*Manganese peroxidases* – These are extracellular enzymes like LiP, and both use heme as a cofactor. The primary difference between manganese peroxidase (MnP, E.C. 1.11.1.13) and LiP is that MnP requires Mn^2+^ for its redox reactions. The catalytic cycle of MnP is like that of LiP, generating the enzyme intermediates MnP1 and MnP2. However, MnP only functions in the presence of Mn^2+^. Without Mn^2+^, MnP cannot be reduced back to its active form. The catalytic mechanism involves the oxidation of Mn^2+^ to Mn^3+^, which then oxidizes other organic substances. In terms of catalytic reaction types, MnP and LiP exhibit differences (Chowdhary et al. 2019). MnP can catalyze not only oxidation reactions (such as C–C cleavage, alkyl-phenyl cleavage, and oxidation of benzyl alcohols), but also reduction reactions (such as the oxidation of hydroquinones by Mn^3+^ to form semiquinone radicals that can act as reductants). In the presence of Mn^2+^ and O_2_, MnP can oxidize some substrates even without H_2_O_2_. Combinatorial chemical and enzymatic treatments can enhance the depolymerization of subbituminous coal resulting in chemically heterogeneous and complex liquefaction products including humic and fulvic acids (Huang et al. 2013). Klein et al. also reported that adding Mn^2+^ to the culture medium enhanced the solubilization of brown coal by white rot fungi (Klein et al. 2014).

*Esterases* – In our perspective on the development of a phytoremediation strategy for the sustainable rehabilitation of land disturbed or degraded by coal mining we summarised the current available information on the role of esterases in coal liquefaction (Sekhohola-Dlamini et al. 2022). Esterases are hydrolase enzymes that use water as a co-substrate and by hydrolysis, convert esters into acid and alcohol residues. Although early studies on the bacterial and fungal biodegradation of coal suggested the action of an esterase-like activity no real evidence for its involvement in coal solubilization was available. However, results from later studies using cultures of *Penicillium decumbens* P6 as the catalyst confirmed that an esterase had been partially responsible for conversion of lignite to low-molecular-mass humics containing less aromatic carbon but higher amounts aliphatic carbon (Yang et al. 2018a, b). Even so, caution in the interpretation of these findings is needed. Sudheer et al. (2016) indicate that steric hindrance may prevent the enzyme from reaching active sites in the coal molecule and hinder esterase-induced hydrolysis. Clearly, this is an area of the coal biosolubilization process that is deserving of more in depth study as it was recently shown that coal biodegradation by *Pseudomonas japonica* increased when an esterase was added together with rhamnolipid, a biological surfactant (Shi et al. 2023).

*Monooxygenases* – Monooxygenases initiate the oxidation process by introducing oxygen and may operate similarly when supplied coal as a substrate. Support for this has emerged from studies on the removal of sulfur from coal by a desulfurizing strain of *Streptomyces* isolated from oil-contaminated soil (Reddy and Rao 2023). These authors show that by breaking the carbon–sulfur (C-S) bond and by converting dibenzothiophene (DBT) in coal to its oxide, S is successfully mineralized by *Streptomyces* sp.VUR PPR 102. The four-step reaction is catalyzed by the enzymes DszA, B, and C. The genes *dszA*, *dszB*, and *dzsC* (i.e., dsz operon) encode the enzyme proteins DszA, DszB, and DszC, respectively. Of these proteins, DszC, a DBT monooxygenase, catalyzes the first of two consecutive reaction steps viz., conversion of DBT to DBT-oxide (DBTO) and then DBTO to DBT-sulfone (DBTO2). In the third step, DBTO2 is converted to hydroxyl phenyl benzene sulfonate (HPBS) by the DszA protein, a DBTO2 monooxygenase. The DszB enzyme (HPBS desulfinase) catalyzes the conversion of HPBS to 2-HBP and sulfite (Reddy and Rao 2023). In another example of monooxygenase involvement in bioremediation, microbial degradation of the chemical pollutant 2,6-dimethylphenol (DMP) common in groundwater and coal chemical wastewater was used to probe and identify the responsible activity. Thus, Ji et al. (2022) characterized MpdAB, produced by the DMP-degrading bacterium *Mycobacterium neoaurum* B5-4, as 2,6-dimethylphenol monooxygenase, a two-component flavin-dependent monooxygenase. Transcription of the parent genes of the MpdAB protein, *mpdA* and *mpdB*, was substantially increased upon exposure to 2,6-DMP. It remains unknown whether other monooxygenases participate in the microbial degradation of coal. Nevertheless, a major oxidative detoxification catalyst responsible for removing many hydrophobic pollutants from the environment is cytochrome P-450 (CYP). Several variants of this monooxygenase have been isolated and characterized and roles in hydrocarbon biodegradation by fungi and bacteria have been considered. Some discussion on the potential involvement of selected bacterial CYP in coal biodegradation including CYP101 from *Pseudomonas putida* and CYP102 from *Bacillus megaterium* has been offered (Olawale et al. 2020) and more detailed perspectives are available (Urlacher and Girhard 2019).

*Dioxygenase* – Dioxygenases catalyze the cleavage of aromatic rings, while cyclooxygenase play a critical role in opening stable aromatic ring structures. Singh and Tiwary (2017) isolated *Pseudomonas stutzeri* P2 from coal mines which exhibited a capacity to degrade high concentrations of both high molecular weight polycyclic aromatic hydrocarbons (PAHs) like pyrene and low molecular weight PAHs such as phenanthrene. Polymerase chain reaction (PCR) amplification with specific gene primers revealed the presence of PAH-ring hydroxylating dioxygenase (PAH-RHD) and catechol 2,3 dioxygenase (C23O) and catechol 1,2-dioxygenase (C12O) indicating the involvement of various dioxygenase enzymes in the PAH degradation process by this bacterial strain. Furthermore, this bacterium demonstrated an ability to metabolize a variety of PAHs including phenanthrene, naphthalene, pyrene, and fluorene and other coal-derived hydrocarbons such as benzene, toluene, ethylbenzene, xylene, and carbazole (Singh and Tiwary 2017). This strain of *Pseudomonas* was also able to produce biosurfactants that increased the emulsification index and reduced the surface tension of the medium, indicative of a role for biosurfactants in PAH degradation. Together, this information suggests that the combination of genes coding for dioxygenase activity coupled with biosurfactant production facilitated the conversion of coal into simpler, metabolizable compounds, enabling microorganisms to utilize coal as a carbon source.

*Alkaline proteases* – These enzymes initiate the formation of reactive oxygen radicals which facilitate the oxidation of phenols and alcohols to esters in coal (Pawar et al. 2023). Additionally, alkaline proteases engage in reactions to cleave alkyl side chains from the coal structure (Bielińska et al. 2018). Shi et al. (2024) found that during the degradation of Heishan coal, which is a form of coal rich in oxygen-containing functional groups, alkaline proteases secreted by *Bacillus* sp. XK1 could depolymerize this substrate to produce long-chain alkanes and aromatic hydrocarbons, esters, and other small molecules. Similarly, Sabar et al. (2019) reported that alkaline proteases released by an indigenous fungal isolate AD-1, identified as *Rhizopus oryzae*, isolated from a coal mine in Pakistan, were able to hydrolyze the alkyl side chain of coal. The black coal-derived liquid contained aromatic acids, fatty acids, alkanes, amines, and amides. Thus, the indigenous AD-1 fungal strain sourced from a coal environment was able to decarboxylate, deaminate, and cleave side chains of aromatic rings demonstrating its ability to biodegrade low rank coal.

## Anaerobic biodegradation

Fermentative metabolism illustrates how important the anaerobic process and its associated microorganisms are for energy extraction from coal. Genes that encode hydrolases and biosurfactants are pivotal in the early hydrolytic reactions of anaerobic fermentation, while hydrogenases are essential for H_2_ production and utilization. Additionally, genes expressed by methanogens and acetogens, such as the *mcrA* gene in methanogenic archaea, are instrumental in converting coal-derived compounds into CH_4_ and CO_2_. It is the products of expression of these functional genes that facilitate coal breakdown in the absence of oxygen and contribute to the anaerobic production of the energy feedstock CH_4_.

All evidence to date indicates that anaerobic biodegradation of coal proceeds through hydrolytic and fermentative metabolism in which coal-derived compounds are converted into organic acids, alcohols, and CH_4_ and CO_2_ (Orem et al. 2010; Gupta and Gupta 2014; Iram et al. 2017; Gong et al. 2022). Thus, the processes remain a sequence of reactions, with initial substrates being metabolized to intermediate products, which are then further processed by methanogens and other anaerobes. Such fermentative pathways are used by microorganisms to extract energy from a substrate in the absence of oxygen. It is the functionality of these steps that facilitates the breakdown of a complex and recalcitrant material like coal in the absence of oxygen and, which fuels its bioconversion.

Anaerobic bioconversion begins with hydrolysis of large precursor macromolecules via a series of fermentative reactions that culminate in methanogenesis. Three main pathways are used for CH_4_ production; 1) Hydrogenotrophic methanogenesis which involves the reduction of CO_2_ to CH_4_ using H_2_, 2) Methylotrophic methanogenesis which uses methylated compounds, such as methanol, to produce CH_4_, and 3) Acetoclastic methanogenesis involving the breakdown of acetate into CH_4_ and CO_2_.

All methane-producing bacteria express the *mcrA* gene which encodes the α-subunit of methyl-coenzyme M reductase (MCR, EC 2.8.4.1). This Ni-dependent enzyme catalyses the reversible reduction of methyl-coenzyme M (CH_3_–S–CoM) with coenzyme B (H–S–CoB) to form CH_4_ and the heterodisulfide, CoM–S–S–CoB, as indicated below.

$$CH_{3} - S - CoM \, + \, H - S - CoB \, \leftrightarrow \,CH_{4} + \, CoM - S - S - CoB$$

Almost all species of methanogenic Archaea oxidize H_2_ and use CO_2_ as an electron acceptor. Species from several families can also use formate, methanol, methylamine, and acetate. Although the routes to CH_4_ can be quite different, they all end with its formation as a waste or by-product of respiratory activity. The concomitant production of the heterodisulfide is much more important for survival, so most methanogens couple the reduction of CoM − S − S − CoB indirectly to ATP synthesis. Since methanogenic Archaea are not monophyletic, population composition and community abundances based on 16S rRNA analysis as the marker gene is limited. Thus, and perhaps not surprisingly, the above suite of chemical interactions has pivoted expression of the *mcrA* gene as a better tool for analysing biodiversity changes in methanogenic communities than 16S rRNA.

Hydrolysis and acidogenesis are the stages that involve a wide range of heterotrophic species: exclusively or optionally anaerobic, in which a mixture of volatile fatty acids (VFA), acetate, lactate, propionate, butyrate, etc., neutral compounds (ethanol), gases (CO_2_ and H_2_), and ammonium are produced. These bacteria frequently have a generation phase shorter than that of the biocatalysts responsible for subsequent reactions in the anaerobic process. Therefore, hydrolytic and acidogenic action can lower pH and inhibit the development of populations required for later stages. Acetogenesis arises from the oxidative hydrolysis of metabolites formed in the preceding stages e.g., organic acids and alcohols, and is carried out by proton-reducing bacteria that form H_2_ and acetate as products in a series of reactions as follows:

$$lactate \, + \, H_{2} O\, \leftrightarrow \,acetate \, + \, 2H_{2} + \, CO_{2} \left( { + 4.18 \, kJ/mole} \right)$$

$$ethanol \, + \, H_{2} O\, \leftrightarrow \,acetate \, + \, 2H_{2} \left( { - 9.6 \, kJ/mole} \right)$$

$$butyrate \, + \, H_{2} O\, \leftrightarrow \,acetate \, + \, 2H_{2} \left( { - 48.1 \, kJ/mole} \right)$$

$$propionate \, + \, H_{2} O\, \leftrightarrow \,acetate \, + \, 3H_{2} + \, CO_{2} \left( { - 76 \, kJ/mole} \right)$$

Acetoclastic methanogens are members of the Methanosarcinales (e.g., *Methanosarcina* and *Methanosaeta*) and like others, metabolize acetate to derive energy as follows,

$$CH_{3} COOH\, \to \,CO_{2} + \, CH_{4}$$

Acetate dismutation is a type of fermentation but unlike typical fermentation pathways, ATP synthesis during methanogenesis is based on electron transport-linked phosphorylation, not substrate-level phosphorylation. Some acetoclastic Methanosarcinales and at least one member of the Methanomicrobiales use a second pathway termed methylotrophic methanogenesis, in which methanol or methylamines serve as substrates. Acetoclastic methanogenesis seems to be most active in freshwater sediments, stored sludges/slurries and in anaerobic digestors, where acetate contributes roughly two-thirds of the carbon for CH_4_ formation. Methylotrophic methanogenesis involves the activation of methylated compounds such as methanol and trimethylamine via substrate-specific proteins that transfer the methyl group to methanogenesis via CH_3_-S-CoM. This form of methanogenesis is important in anoxic environs where methylated substrates abound. A third route to CH_4_, hydrogenotrophic methanogenesis, occurs in all five orders of known methanogens viz., the Methanosarcinales, Methanococcales, Methanomicrobiales, Methanopyrales, and Methanobacteriales and uses H_2_ to reduce CO_2_ (or CO or formate) according to the following equilibrium.

$$4H_{2} + \, CO_{2} \, \to \,CH_{4} + \, 2H_{2} O$$

Well-studied organisms that produce CH_4_ via H_2_/CO_2_-methanogenesis include *Methanosarcina barkeri*, *Methanobacterium thermoautotrophicum*, and *Methanobacterium wolfei*. While the process is a type of respiration and the organisms that use it grow autotrophically, a typical electron transport chain is not present. Rather, methanogens rely on several special coenzymes including F_420_, which is involved in the activation of H_2_, and coenzyme M which is involved in the terminal reduction of CH_3_-groups to CH_4_.

**Fig. 3:**
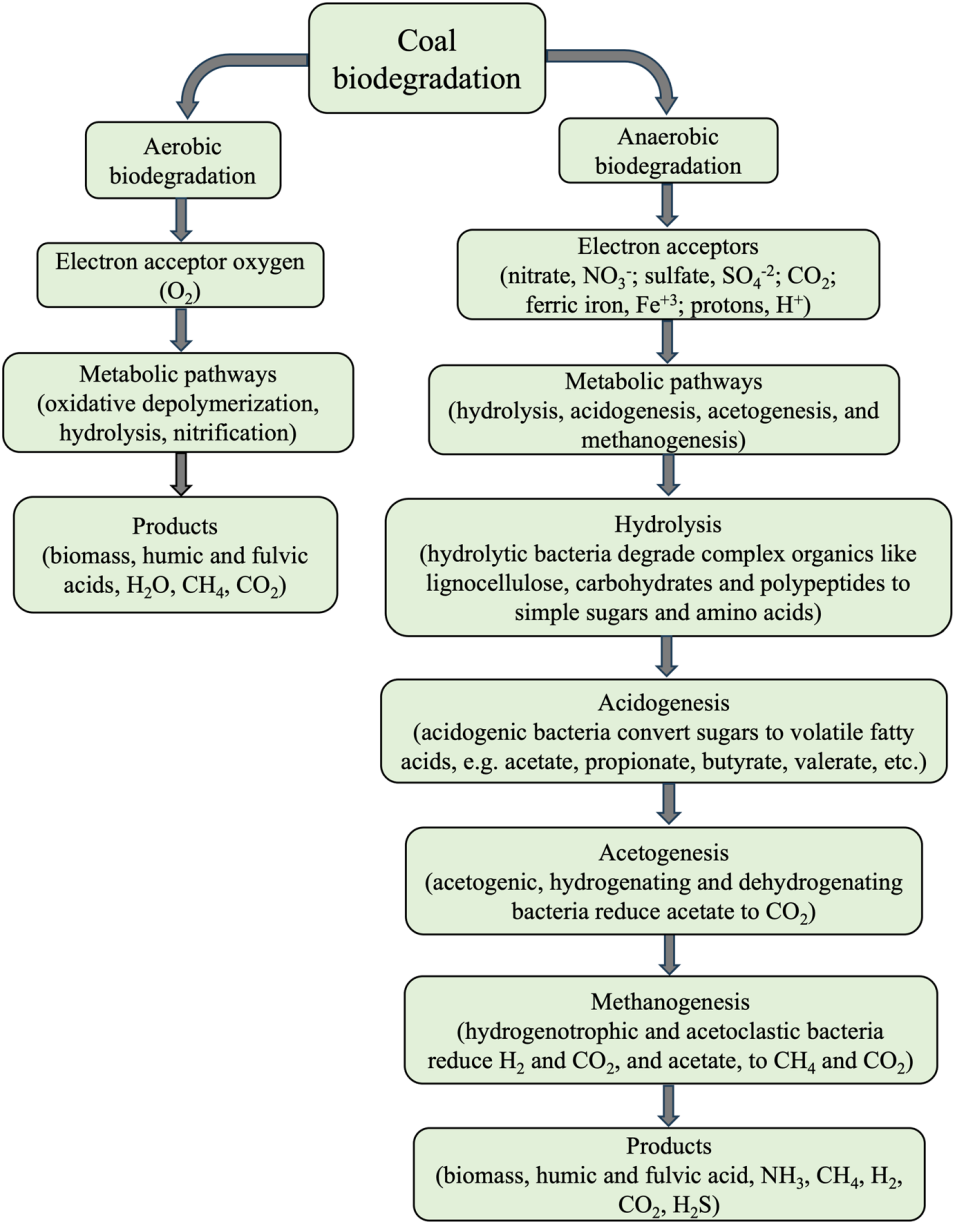
Sequence of reactions considered typical for the anaerobic biodigestion of coal in comparison to the probable mechanistic processes involved in the aerobic biodegradation

Although the energetics of hydrogenotrophic methanogenesis seem favourable theoretically, in practice, cell growth rates and yields are lower than can be predicted. This is partly due to autotrophic growth using some of the substrate for C-assimilation, including a significant amount of ATP. Acetoclastic methanogenesis is energetically less favourable, resulting in slow growth rates and low cell yields. Compared to sulfate reducing bacteria, methanogens are inferior competitors for both substrates, H_2_ and acetate. Thus, when sulfate is present, methanogenesis is usually low. However, in sulfate-depleted anaerobic habitats, methanogens play a central role as H_2_-scavengers and in the terminal mineralization of acetate. As such, they contribute significantly to the global carbon cycle. The various ways organic matter is biodegraded under anaerobic conditions, in contrast to aerobic processes, is outlined in Fig. 3.

Hydrolytic microorganisms are required for anaerobic coal biodegradation to proceed, and the efficiency of hydrolysis directly impacts the process. These microbes release extracellular enzymes that target macromolecular PAH and convert these into simple organics that can be more easily assimilated while providing substrate for subsequent stages in the anaerobic process. And a recent study showed hydrolysis to be the rate-limiting step in the anaerobic fermentation of coal, with products being mainly aromatic compounds and organic acids (Xia et al. 2021). Thus, strains of *Bacillus polymorpha* and *Bacillus sphaericus*, that were extracted from long-flame coal, have been validated as highly effective hydrolytic bacteria to aid the anaerobic conversion of coal to CH_4_. Other efficient hydrolytic bacteria can be obtained by supplementation with exogenous microflora (e.g., AS) that appears to promote the transformation and humification of low rank coal (Kozhakhmetova et al. 2024). Chemical pre-treatments, such as NaOH, also enhance bioavailability by altering the coal’s structure and increasing the solubility of organic carbon (Guo et al. 2020b). Additionally, supplementing with nutrients such as nitrogen, phosphorus, and trace elements enhances microbial activity and coal breakdown (Kozhakhmetova et al. 2024). Co-digesting with materials rich in organic carbon, like straw biomass also increases the H/C ratio to improve CH_4_ production (Khan et al. 2024b; 2024c).

Whereas carbohydrates are typically reduced to monosaccharides such as glucose and fructose by glycoside hydrolases, the depolymerization of coal-containing PAH involves a hydrolase encoded by the *bam*A gene, which plays a crucial role in ring-cleaving processes (Ruan et al. 2016). Li et al. (2023a) investigated the role of the *bam*A gene product in the anaerobic degradation of aromatic compounds in coal and found benzoyl-CoA to be the most common intermediate formed during its anaerobic degradation. These authors also showed that the *bam*A gene product is the hydrolase responsible for the ring cleavage of 6-oxocylcohex-1-ene-1-carbonyl-CoA in the benzoyl-CoA pathway. Zhang et al. (2022b) found that expression of an enolase (K01689), a hydrolase that can act on 3-phospho-D-erythronate, increased during anaerobic coal biodegradation. In addition, genes coding for the biosurfactants esterase hydrolase enzyme (*estAB*), lichenysin synthetase (*lchA*), and surfactin synthetase (*srfAB*) were also detected with the highest gene abundance in the FG-L coal seam (Schweitzer et al. 2022). Together and in contrast to the aerobic degradation of coal (Fig. 3), these findings provide strong support for operation of hydrolytic bacteria in the initial stages of the anaerobic degradation of coal.

For the acidogenesis phase of anaerobic coal biodegradation, products of the hydrolysis of coal appear to be absorbed by microorganisms and further converted into simple organic compounds (such as VFA, H_2_, CO_2_, alcohols, etc.). And the acid-producing metabolic pathways seem to play a crucial role in determining the overall efficiency of the anaerobic bioconversion of coal. Zhang et al. (2022c) found that a combination of coal and corn straw served as ideal co-substrates. These authors found that this mixture promoted the enrichment of key enzymes involved in acidogenesis (production of organic acids). Additionally, both hydrogenotrophic and methylotrophic methanogenic pathways, which are critical for CH_4_ formation, were likewise elevated. The study also showed that some of the acidogenic enzymes were closely related to carbohydrate-active enzymes (CAZy), a group of enzymes known for degrading complex carbohydrates. These CAZy decompose, (re)-synthesize, and modify complex carbohydrates including glycans and various glycoconjugates (Sun et al. 2023). According to their function, they are divided into auxiliary activities, carbohydrate-binding modules, carbohydrate esterases, glycoside hydrolases (GHs), glycosyl transferases, and polysaccharide lyases. The genes encoding for GHs are present in most genomes and correspond to almost half of the enzymes in the CAZy group and are essential in many biotechnology applications (Cantarel et al. 2009). Pyruvate, an important intermediate in the acid-producing pathway, is further converted to a variety of products such as acetate, propionate, butyrate, ethanol, propanol, butanol, H_2_, and CO_2_ (Chen et al. 2013).

Methanogenesis is the final phase in the anaerobic biodegradation of coal and is dominated by formation of CH_4_. For the acetoclastic pathway of CH_4_ production, the key enzymes involved catalyze the conversion of acetic acid to acetyl-CoA and include acetyl-CoA synthetase or acetate-CoA ligase (EC 6.2.1.1), acetate kinase (EC 2.7.2.1), and phosphate acetyltransferase (EC 2.3.1.8) (Yin et al. 2018). Acetyl-CoA synthetase, an acidic mercaptan ligase, forms C‑S bonds and acts on propionate and acrylate while acetate kinase, phosphorylates ADP using acetyl-P to form ATP and acetate and is an important step in energy conservation during the acid-producing stage. Phosphate acetyltransferase catalyzes the phosphorylation of acetyl-CoA to form acetyl phosphate. Another key acetoclastic pathway enzyme is acetyl-CoA carboxylase (ACS, EC 6.4.1.2), responsible for the carboxylation of acetyl-CoA to malonyl-CoA to initiate fatty acid synthesis, is a nickel-containing enzyme which together with carbon monoxide dehydrogenase (CODH), forms carbon monoxide dehydrogenase/acetyl-CoA synthase (CODH/ACS). This five-subunit enzyme is present in archaea and other anaerobic bacteria (Lindahl 2004) and is primarily involved in the conversion of CO_2_ to acetyl-CoA and generally referred to as CO-methylating acetyl-CoA synthase. The beta subunit of the CODH/ACS complex is produced by *Methanosarcina* and *Methanoculleus* (Ma et al. 2020) and has been confirmed as an acyltransferase involved in the synthesis of acetyl-CoA from CO_2_/H_2_. Formylmethanofuran dehydrogenase subunit A, an oxidoreductase that acts on aldehyde or oxygen groups of donors with ferritin as a receptor, participates in CH_4_ production from CO_2_ via the oxidation of coenzyme M to CO_2_. The alpha subunit of the coenzyme F420 hydrogenase is also considered a type of oxidoreductase that can bind to other known receptors with hydrogen as donor. Tetrahydromethanopterin S-methyltransferase subunit A, a kind of methyltransferase, transfers carbon-containing groups and participates in the conversion of CO_2_ to CH_4_. In addition, methyl-coenzyme M reductase alpha subunit transfers sulfur-containing groups and catalyzes the last step in CH_4_ biosynthesis by methanogens. In addition, *Methermicoccus* methanogens produce CH_4_ from more than 30 various methoxylated aromatic compounds (MACs) as well as from MAC-containing coals (Mayumi et al. 2016). Furthermore, the *AmaM* genes encoding acetyl-CoA synthesis, acetyl-CoA oxidation, and CO_2_-reducing methanogenesis in the methanogen *Methermicoccus shengliensis* have been shown to be key in the anaerobic conversion of coal to CH_4_ (Mayumi et al. 2016). Together, the accumulated evidence indicates that various types of methanogenesis appear to be operative, which suggests many distinct methanogenic processes pointing to multiple organisms participating in the overall anaerobic bioconversion of coal.

## Microbial molecular and genetic studies on coal biodegradation

In recent years, the application of genetic engineering to enhance the innate biodegradation capability of microorganisms has gained significant attention (Xiang et al. 2021). Techniques involving recombinant DNA and RNA, as well as genetically modified organisms (GMO), have been effectively employed in biodegradation initiatives. Alterations to microbial genes have resulted in the development of novel metabolic pathways that augment bioremediation processes (Furukawa 2003; Pieper and Reineke 2000). As discussed above, certain fungi and bacteria possess the ability to produce enzymes that degrade lignin, a principal component of coal. By upregulating the expression of these genes, microbial strains can be engineered to degrade coal more effectively (Kozhakhmetova et al. 2024). Moreover, genetic modifications can optimize the metabolic pathways of microbes, enabling them to process coal-derived compounds and produce valuable products such as biofuels more efficiently. Perhaps the best example to date of the successful manipulation of a gene considered to play a vital role in coal biosolubilization was the expression of a *Fusarium oxysporum* LAC in *Pichia pastoris*, which once expressed, resulted in the release of humic and fulvic acids from the coal substrate (Kwiatos et al. 2018; 2020).

While enzymes like LiP, MnP and oxidases such as LAC are considered the dominant catalysts in the fungal bioconversion of coal, proteomic analysis during the biodegradation of lignite by *Fusarium* sp. NF01 has revealed altered (upregulated and down regulated) expression of 62 proteins, some of which were catalysts of coal degradation (Niu et al. 2022). Niu et al. (2022) argue that secretome analysis should be inclusive and focus on integral enzymes expressed as part of the response mechanisms to better understand the overall biodegradation of coal.

For the prokaryotes, understanding natural microbial communities is essential for the further development of synthetic microbial consortia (Nwankwegu et al. 2022). A complex mixture of hydrocarbons, including n-alkanes, aromatics, and PAH was successfully biodegraded using *Acinetobacter baumannii* S30, which was genetically modified by incorporating the *lux* gene, a reporter gene that encodes luciferase, a protein that emits light (Zakaria et al. 2021). This modification allowed for creation of the recombinant strain *Acinetobacter baumannii* S30 pJES, which was used to monitor progression of bioremediation and survival of the engineered strain. Furthermore, the model bacterium *Streptomyces coelicolor* M145 was modified to over express the enzyme alkane monooxygenase, encoded by the *alkB* gene, resulting in the efficient degradation of hexadecane. Similarly, the strain *Acinetobacter sp.BS3*, recognized for its alkane-degrading capabilities, exhibited enhanced degradation of n-alkanes and aromatic hydrocarbons following modifications with the *xylE* gene, which encodes the enzyme catechol 2,3-dioxygenase (Xiang et al. 2021).

Additionally, the discipline of synthetic biology augments the potential for coal conversion by facilitating the design of novel metabolic pathways and regulatory systems. This methodology enables the formation of microbial consortia, wherein each microorganism is engineered to fulfill a specific function in the coal transformation process (Xiang et al. 2021). For example, one study illustrated the efficacy of bioaugmentation using activated sludge to enhance microbial degradation of low-rank coal. The incorporation of activated sludge markedly increased the metabolic activity of the coal microbiota, resulting in elevated production of humic acids and other valuable products (Kozhakhmetova et al. 2024). Nonetheless, several challenges persist in the application of these technologies. The heterogeneous nature of coal complicates the development of microbial strains capable of efficiently degrading all its components (Qin et al., 2022). Furthermore, environmental and safety concerns regarding the release of genetically engineered microbes must be meticulously addressed. It is imperative to ensure that these microbes do not exert unintended adverse effects on ecosystems to ensure their safe and effective utilization(Xiang et al. 2021).

## Coal-degrading biocatalysts

### Coal-degrading eukaryotic organisms

Coal-degrading eukaryotic organisms identified to date comprise largely fungi (Sekhohola et al. 2013; Akimbekov et al. 2022). However, several lines of investigation suggest the possibility that other eukaryotes such as microalgae, blue-green algae, and higher macrophytes have the potential to contribute to the biotransformation of coal and presumably, its biological degradation.

An early and still uncorroborated study suggested the presence of microalgal species in brown coal from Sokolov, Czechia and in lignite from mines in Cottbus, Germany (Lukešová 2001). More than 100 species were identified including green algae, cyanobacteria, and diatoms. No detail on the growth and/or utilization of brown coal/lignite as a carbon source by any of these algae was provided and there does not appear to have been any follow-up study. While the potential of microalgae from coal mine water for CO₂ sequestration and biodiesel production are being actively explored (Anahas et al. 2025), coal microparticles seem to impact marine and freshwater organisms and in particular algae (Ahrens and Morrisey 2005; Tretyakova et al. 2021) and, cyanobacteria from marine, freshwater, and terrestrial habitats produce CH_4_ which seems to indicate that methanogenesis at least in these organisms, is associated with primary productivity (Bižić et al. 2020). Since many cyanobacteria exhibit heterotrophic and mixotrophic growth, this source of biogenerated CH_4_ may be linked to the assimilation of complex carbon and/or the conversion of methylated intermediates that arise from degradation of dissolved and suspended organics including coal particles.

In 2022, it was reported that fungi inhabiting the sediments below the ocean floor were capable of anaerobically degrading PAHs and that biodegradation positively correlated with the anaerobic growth of fungi (ul Arifeen et al. 2022). Fungal biodegradation of PAHs was not related to ligninolytic activity but rather to action of carboxylases implying that these organisms survive long periods of anoxia by utilizing PAHs as a source of carbon and thus play a significant role in global carbon cycling.

Another potential contributor to coal biodegradation includes the macrophytes that are used in subsurface flow constructed wetlands. Wang et al. (2022a) posed the question, can mine waste be used as substrate in constructed wetlands to intensify nutrient removal? In their study, coal gangue, iron ore and manganese ore were used as substrates in a 385-day wastewater treatment experiment. Results showed that mine waste increased removal of N and P, enhanced biofilm establishment but with increased risk of heavy metal-induced oxidative stress to plants. Another study showed successful establishment of *Phragmites australis*, better macrophyte productivity measured as PSII quantum yield, and greater nutrient removal from wastewater in a horizontal subsurface flow constructed wetland with a filter bed of coal discard (Tebitendwa and Cowan 2023). Ultimate analysis of the filter bed residue indicated that changes in elemental C and N content were not due to leaching but a consequence of macrophyte growth, rhizosphere microbiome establishment, shoot recruitment, and nutrient abstraction. Research has confirmed that arbuscular mycorrhizal fungi (AMF) colonize most wetland plants and participate as symbionts and as biocatalysts in phytoremediation of different substrates (Hu et al. 2021). It is no surprise therefore that fungi occur naturally in mine soils, in coal reservoirs and participate both directly and indirectly in coal bioconversion and coal product assimilation (Sekhohola et al. 2013; Sekhohola-Dlamini et al. 2022; Akimbekov et al. 2022).

Fungi seem to possess an ability to cleave larger aromatic complexes into smaller structures and molecules that are amenable to further metabolism by lower microorganisms such as bacteria and archaea. Fungi may also be of value as biocatalysts in the production of value-added products, and in the development of coal biodegradation technologies. For instance, the critical biocatalytic role of fungi in a batch reactor bioprocessing of waste coal has recently been reported (Ahmed and Sharma 2021). Initial biosolubilization of coal reject by a fungal consortium, was subsequently followed by inoculating the same bioreactors with sludge from a food waste anaerobic digester that led to production of CH_4_ and volatile fatty acids (Ahmed and Sharma 2021). Fungal catalysts in the production of humic substances, which benefit land rehabilitation, soil restoration and agricultural production have also attracted attention in research (Cowan et al. 2016; Sekhohola and Cowan 2017; Sekhohola-Dlamini et al. 2022). Thus, significant production of humic acids from fungal bio-liquefaction of coal, with extracted yield of 67.4 ± 4.3 percent (w/w), has been achieved (Ghani et al. 2021). And optimization of process parameters for bioconversion of lignite by the Ascomycota *Hypocera lixii* WF8 by an artificial neural network combined with a genetic algorithm has been attempted and models developed and used to understand release mechanism of humics during the bioconversion of lignite (Yao et al. 2021). Fungal isolates, mostly of the phyla Ascomycota, Basidiomycota, and Zygomycota are continuously reported as catalysts in the solubilization of coal of different ranks, where the by-products are predominantly humic substances (see studies in Table 2). Another important coal biodegradation technology, bioleaching of coal using fungal catalysts for clean coal technology, has also been explored (Manoj and Elcey 2013; Kwiatos et al. 2018).

**Table 2:** Fungal strains that have been identified as coal degraders and the products formed from coal degradation (*studies published from 2013 to 2024*)

Phylum	Genus	Coal type	Catalyst(s)	Product(s)	Source	Refs
Ascomycota	*Penicillium aculeatum 13–2-1*	weathered coal		humic acids	rhizosphere of *Zelkova serrata*	Ren et al. 2023
Not specified	Isolates M13 and MI	lignite	cellulase, xylanase	humic acids	Pakistani Thar lignite	Ghani et al. 2022
Ascomycota	*Hypocrea lixii *HN-1	bituminous		humic acids	coal mine soil	He et al. 2023
Ascomycota	*Aspergillus flavus* NF-2	lignite		humic acids	coal mines	Rehman et al. 2022
Ascomycota	*Trichoderma citrinoviride A-1*	lignite	oxidase, lignin peroxidase, laccase	Possibly organic acids	seeds of white Tremella	Feng et al. 2021
Ascomycota, Basidiomycota	*Trichoderma viride*,* Neurospora discreta* *Phanerochaete chrysosporium*	sub-bituminous	laccase	methane, volatile fatty acids	Subabul wood tree, National Collection of Industrial Microorganisms	Ahmed and Sharma 2021
Ascomycota, Zygomycota	*Aspergillus tubingensis*, *Purpureocillium lilacinum, Simplicillium subtropicum, Penicillium daleae, Trichoderma koningiopsis* *Mucor circinelloides, Cunninghamella bertholletiae,*	sub-bituminous	laccase	humic acids, fulvic acids	Coal rhizospheres of *Ageratum conyzoides*, *Axonopus compressus*, *Emilia coccinea*, *Synedrella nodiflora*, *Urena lobata* and *Sida acuta*	Nsa et al. 2022
Ascomycota	*Trichoderma strains*	lignite	laccase	solubilisate of water-soluble polymer (humics)	lignite and rotten wood bark	Smoleňová et al. 2020
Zygomycota	*Rhizopus oryzae* AD-1	low rank coal		organic black liquid (humics)	low rank coal	Sabar et al. 2019
Ascomycota	*Fusarium oxysporum*	lignite	laccase	humic acids, fulvic acids	brown coal	Kwiatos et al. 2018
Ascomycota, Basidiomycota	*Trichoderma sp. AY6,* *Phanerochaete sp. AY5*	not specified		not specified		Malik et al. 2016
Basidiomycota	*Trametes hirsuta, Trametes maxima*	lignite	manganese peroxidase, lignin peroxidase	not specified		Klein et al. 2014
Ascomycota	*Penicillium chrysogenum* MW1	low rank coal		not specified	core sample of coal	Haider et al. 2013
Ascomycota	*Aspergillus niger, Aspergillus flavus, Penicillium spp*	low rank coal	Not specified	organic acids	Not specified	Manoj and Elcey 2013
Ascomycota	*Trichoderma strains*	lignite	laccase	solubilisate of water-soluble polymer (humics)	lignite and rotten wood bark	Smoleňová et al. 2020
Zygomycota	*Rhizopus oryzae* AD-1	low rank coal		organic black liquid (humics)	low rank coal	Sabar et al. 2019

Fungal species that occur naturally in coal environments are believed to utilize coal as a source of energy (Sharma and Sumbali 2019). Over the years and as shown in Table 2, various fungal isolates capable of coal degradation have also been identified from woody and soil ecosystems. Some fungal species from the plant rhizosphere have also been found to contribute directly to coal bioconversion. One well-studied example is *Neosartorya fischeri*, a member of the Aspergillus group, which was identified from rhizospheric soil of the grass *Cynodon dactylon* and shown to oxidatively degrade coal discard leading to an increase in humic-like substance concentration and formation of a soil-like material (Sekhohola and Cowan 2017); a phenomenon later corroborated by Nsa et al. (2022). Coal-degrading fungi belonging to many taxonomic groups including *Ascomycetes*, *Zygomycetes*, *Sordariomycetes*, and *Leotiomycetes* have been detected in the rhizosphere soil of plants growing in a sub-bituminous coal landfill (Nsa et al. 2017). Similarly, it has been reported that lignite coal is more susceptible and can be easily dissolved by white rot fungi such as *Trichoderma atroviride*, *Phanerochaete chrysosporium*, and *Penicillium* species such as *Penicillium chrysogenum*. Moreover, Detman et al. (2018) discovered that detritic lignite is more vulnerable to fungal attack.

While many studies do not specify or identify the catalysts, it has been reported that non-enzymatic activity including alkaline metabolites, chelators and surfactants are also involved in the fungal bioconversion of coal (Sekhohola et al. 2014; Kwiatos et al. 2018; 2020; Nsa et al. 2022; Akimbekov et al. 2022). In fact, a recent study on the biosurfactant activity of the fungus *Hypocrea lixii* AH established that the hyphae, spores, and EPS of this fungus modified the surface of lignite coal much like biosurfactants (He et al. 2023). Of the fungi that have been found to exhibit coal biodegradation activity (Table 2), most appear to transform coal substrate into PAH, monocyclic aromatics, heterocyclic nitrogenous compounds, and aliphatics (Akimbekov et al. 2022). But perhaps the most important aliphatic produced in the biotransformation of coal and waste coal is CH_4_.

In contrast to the soil environment, coal seams are anaerobic and access by aerobic microbes is constrained. Instead, it is more feasible that anaerobic fungi can survive in coal seams and colonize and use ‘seam’ coal as a carbon source. Thus, it is not surprising that species of facultative anaerobic fungi including *Aspergillus, Fusarium, Rhodotorula, Trichoderma, Shizophyllum* and *Mortierella* detected in the Qinshui Basin, a critical frontier of CBM exploration and development in China, are able to decompose plant derived lignin-based compounds in coal as well as aromatics (Guo et al. 2017). Other fungal strains of the genera *Thielavia, Humicola, Mortierella, Trichoderma, Acremonium,* and *Alternaria* are also capable of degrading anthracite bio-electrochemically in an anaerobic reactor (Guo et al. 2020a). And in a stirred tank reactor, *Trichoderma atroviride* was used to degrade coal (Oboirien et al. 2013) while *Alternaria* was shown to eliminate sulfur and ash from coal, thus providing favorable conditions for methanogenic bacteria to reduce CO_2_ and formate (Amao 2018; Şener et al. 2018). The coal mine fungal flora designated AD-1 found in low rank coal simultaneously deaminates and decarboxylates this substrate to remove side chains from more complex aromatic compounds (Sabar et al. 2019).

Thus, there continues to be a growing interest in fungi and the ability of these microbes to biosolubilize coal due to their remarkable biodegradation potential. Species of interest include *Fusarium oxysporum, Aspergillus sp., Verticillium sp., Trichocladium canadense* and *Acremonium sp*. which actively degrade PAH under microaerobic conditions (Silva et al. 2009). Others secrete ligninolytic enzymes which act synergistically to biodegrade lignin-containing organic matter (Kumar and Chandra 2020). Furthermore, species of *Candida, Penicillium, Aspergillus, Fusarium,* and *Pichia* are commonly used to eliminate PAH including pyrene, naphthalene, dibenzothiophene, and phenanthrene and degrade the aromatics in low grade coal (Detman et al. 2018; Gong et al. 2022). Thus, *Penicillium brefeldianum, Trametes versicolor, Trichoderma reesei, Aspergillus niger, Aspergillus nidulans, Phanerochaete chrysosporium, Ganoderma lucidum,* and *Trichoderma longibrachiatum* are examples of fungi that possess lignin degrading activity and are candidate species for enrichment for coal biodegradation. *Rhizopus oryzae* also solubilizes coal and, up to 36.8% is degraded nine days after initiation of incubation (Sabar et al. 2019).

## Coal-degrading prokaryotes including bacteria, methanogens, and archaea

Several bacterial species have been isolated and investigated for their ability to biodegrade coal. Under optimal conditions such as standard temperature and pressure, indigenous bacterial species isolated from coal mines, formation water and coal disposal sites, and screened and characterized for coal bioconversion can accomplish maximum coal degradation as they have the ability for fast conversion rates, ease of operation, and short culture times (David et al. 2017; Olawale et al. 2020; Sudheer et al. 2016; Akimbekov et al. 2022). Bacterial species have also been isolated and characterized as autochthonous from lignite mines and assessed for bioremediate potential of coal-overburdened soil (Hamidović et al. 2016; Olawale et al. 2020). David et al. (2017) reported that bacterial strains belonging to the genera *Cupriavidus*, *Pseudomonas* and *Alcaligenes* and isolated from coal sludge, could degrade coal and carry out depolymerization of aromatic compounds. The responsible catalyst was purported to be a LAC-like activity detected using a dye-degradation test. Specific microbial consortia and engineered microbial systems have demonstrated enhanced efficacy of coal degradation. For instance, bioaugmentation with AS has been shown to significantly improve the degradation of low-rank coal (Kozhakhmetova et al. 2024). The microbial consortia present in AS appears to supplant indigenous coal microorganisms and facilitate processes such as sulfur and iron redox transformations, thereby promoting biosolubilization. Furthermore, certain fungi and bacteria, including *Pleurotus ostreatus* and *Geobacter* species, have exhibited a capacity to degrade coal through enzymatic activity. These findings indicate that customized microbial consortia can be developed to optimize the efficiency of coal degradation (Kozhakhmetova et al. 2024). Table 3 lists some of the bacterial strains that have been isolated and identified as coal degraders based on the formation of breakdown products from coal degradation reported to date.

**Table 3:** Bacterial strains that have been identified as coal degraders and products of the coal degradation process (*data from 2013 to 2024*)

Phylum	Genus/species	Coal type	Product(s)	Refs
Actinobacteria, Proteobacteria, Acidobacteria, Chloroflexi, Bacteroidetes, Verrumicrobia, Gemmatimonadetes	not specified	low rank coal	humic substances	Kozhakhmetova et al. 2024
Pseudomonadota	*Stenotrophomonas bentonitica BII-R7*	coal gangue	not specified	Wang et al. 2024
Bacillota	*Bacillus polymorpha, Bacillus sphaericus*	long-flame coal	methane	Xia et al. 2024
Bacillota, Bacteroidota	*Proteiniclasticum, Proteiniphilum, Bacillus, Petrimonas*	lignite	methane	He et al. 2020
Pseudomonadota, Bacillota, Actinomycetota	*Escherichia, Citrobacter, Exiguobacterium, Serratia, Microbacterium*	weathered coal	not specified	Titilawo et al. 2020
Pseudomonadota, Bacillota, Bacteroidota, Firmicutes	*Enterobacter, Acetoanaerobium, Macellibacteroides, Clostridium, Ercella*	anthracite	methane	Guo et al. 2019
Pseudomonadota	*Ochrobactrum cytisi, Novospingobium naphthalenivorans, Alcaligenes faecalis, Pseudomonas fluorescens*	lignite	low molecular products	Kang et al. 2019
Bacillota	*Bacillus sp.* RKB 2	lignite	humic substances	Akimbekov et al. 2019
Pseudomonadota	*Cupriavidus sp, Pseudomonas sp, Alcaligenes sp*	low rank coal	not specified	David et al. 2017
Pseudomonadota, Bacteroidota	*Cupriavidus necator S2A2, Sediminibacterium ginsengisoli S2B14, Sphingomonas sp. S2B18*	low rank coal	low molecular products	Baylon et al. 2017
Bacillota, Actinomycetota	*Aeribacillus, Actinobacteria*	not specified	methane	Barnhart et al. 2016
Bacillota, Pseudomonadota	*Bacillus sp. AY3, Pseudomonas sp. AY2*	not specified	not specified	Malik et al. 2016
Bacillota, Pseudomonadota	*Bacillus mycoides, Acinetobacter baumannii*	lignite	humified organic matter	Cubillos-Hinojosa et al. 2015
Bacillota, Pseudomonadota, Actinomycetota	*Bacillus mycoides, Acinetobacter sp, Enterobacter aerogenes, Microbacterium sp*	lignite	humic acids	Valero et al. 2014; 2016
Bacillota, Actinomycetota	*Bacillus mycoides NS1020, Gordonia alkanivorans S7*	lignite	humic acids	Romanowska et al. 2015

To improve the biodegradation of coal, community-based biodegradation, also referred to as bioaugmentation, is an appealing alternative to individual bacterial strains. Like petroleum and crude oil, coal is a complex organic and difficult to degrade and assimilate as an energy source and growth. However, coal can be easily solubilised by assemblages of microbes working together (Ghazali et al. 2004; Patowary et al. 2016). Different microbial species proliferate on different forms of coal, indicating varying degrees of substrate specialization. Results from studies by Olawale et al. (2020) indicate that coal was degraded by single and mixed microbial consortia including *Exiguobacterium* sp. ECCN21b and *Serratia* sp. ECCN 21b, and this led to 10% and 30% decline in the mass of the coal substrate respectively. *Exiguobacterium* species are known to actively degrade n-alkanes (C9-C26), while *Serratia* species degraded hydrocarbons (Mohanty and Mukherji 2008; Benedek et al. 2011; Olawale et al. 2020). It is distinctly possible that both CO_2_ and CH_4_ are end products of this bacterial bioconversion process.

It is pertinent to point out that terrestrial and aquatic systems actively produce CO_2_ as a product of cellular respiration, and it is increasingly being accepted that these systems also produce CH_4_ and in a manner independent of methanogenic microbes. The first report of terrestrial vegetation actively releasing methane under ambient conditions was by Keppler et al. (2006). Since then, microbial dealkylation of methylphosphonate in marine and freshwater environments has been demonstrated (Repeta et al. 2016; Wang et al. 2017b). While aerobic CH_4_ production appears to be metabolically linked to (bacterio)chlorophyll metabolism and photosynthesis in some species, in members of the Proteobacteria, it occurs due to the degradation of methylphosphonate (Perez-Coronel and Beman 2022). And conversion of methylamine to CH_4_ by *Acidovorax sp*., a genus within the family Comamonadaceae, involves pyridoxylamine phosphate-dependent aspartate aminotransferase (Wang et al. 2021). The latter study also showed that aerobic CH_4_ synthesis can be transferred to other bacteria by introduction into *E. coli* cells of the newly isolated and identified plp-*aat* sequence (codes for PLP-Aat enzyme). Thus, methanogens occur in freshwater and marine environments, cold sediments, hydrothermal vents, as free-living cells, as symbionts with protists and animals, and as symbionts with bacteria that promote anaerobic CH_4_ oxidation. It might therefore be expected that in addition to anaerobic digestion, a suite of genes encoding key enzymes for the aerobic breakdown of coal and its metabolism to CO_2_ and CH_4_ must be present in terrestrial microorganisms competent for biodegradation of the complex coal structure.

Unlike aerobic microbial degradation of coal, anaerobic degradation of complex organic matter is a long-studied and well-documented process carried out by several different bacterial communities which, under appropriate conditions (i.e., at oxidation reduction potential of about -250 mV and pH 7), form stable syntrophic associations. Fermentative respiration by these strict anaerobes typically occurs when a high concentration of organic matter is subjected to extended periods of anaerobiosis such as in primary settlers, slurry ponds, marshes, lake sediment, the gastrointestinal tracts and in buried coal seams. Methane is the product of fermentative growth by these anaerobes, called Archaea, and occurs at low temperature (psychrophilic,10–25 °C) or under mesophilic (30–40 °C) or thermophilic (> 50 °C) conditions. Molecular taxonomic analysis indicates that the Archaea consist of two major subdivisions, the Crenarchaeota and the Euryarchaeota, and one minor ancient lineage, the Korarchaeota.

Coal deposits contain living microbial cells that can break down coal components to generate the substrates necessary for methanogens to produce CH_4_. A review of the organic matter in coal indicated that it consists mainly of aliphatic hydrocarbons, aromatic hydrocarbons, and heteroatom compounds that are converted through specific chemical mechanisms to various forms of dissolved organic matter used to produce CH_4_ (Wang et al. 2018). For instance, acetate is a widely recognized substrate for CH_4_ generation and arises during bacterial degradation of coal in coal habitats and is utilized by acetoclastic methanogens (Krüger et al. 2008). Beckmann et al. (2011) determined that the order *Methanosarcinales* was dominant and that members display acetoclastic (i.e., utilizing acetate as the primary substrate) methanogenesis. *Methanosarcinales* was also identified as the main group responsible for bioconversion of high-quality anthracite coal during the large-scale methane generation process, with *Methanocalculus* sp. as the dominant hydrogenotrophic methanogen (Yang et al. 2018a, b). In addition, several investigations indicate that bacterial populations from CBM are linked to coal biodegradation and solubilization and dominated by communities that belong to *Proteobacteria* and *Actinobacterea* (Akimbekov et al. 2024).

Generally, low to medium rank coals are favorable for biodegradation whereas coals of high rank, have surfaces with low accessibility for bio-extraction (Iram et al. 2017). Additionally, for microbial degradation for CH_4_ generation, coal volatile matter also plays a significant role (Xiao et al. 2013) with high volatile bituminous coal possessing greater CH_4_ production potential than medium volatile bituminous and anthracite coals (Liu et al. 2022). Table 4 lists some of the Archaea that have been isolated and identified as coal degraders based on formation of breakdown products and CH_4_ output in studies published during the period of 2013 to 2024.

**Table 4:** Members of the Archaea that have been identified as coal degraders and the products of the coal degradation process (*studies published from 2013 to 2024*)

Microorganism	Coal type	Product(s)	Refs
Methanosaeta, Methanocella,	anthracite	methane	Guo et al. 2019
Methanothrix, Methanobacterium, Methanolobus, Methanoculleus, Methanobrevibacter, Methanosarcina, Methanofollis, Methanomethylovorans, Methanospirillum, Methanosphaerula	anthracite, bituminous	methane	Su et al. 2018
Methermicoccus shengliensis	bituminous, subbituminous, lignite	methane	Maiyumi et al. 2016
Methanosarcinales, Methanomicrobiales	lignite	methane	Grundger et al. 2015
Methanosaeta, Methanosarcina	subbituminous	methane	Beckmann et al. 2019
Methanomicrobiales, Methanosarcinales	not specified	methane	Wawrik et al. 2012
Methanosarcina	lignite	methane, fatty acids	Yan et al. 2023
Methanosarcina, Methanobacterium, Methanocelleus	lignite	methane	He et al. 2020
Methanoculleus thermophilus	bituminous, subbituminous, lignite	methane	Leavania et al. 2014
Methanolobus, Methanobacterium	not specified	methane	Bao et al. 2019

Methanogenic archaeal communities, however, have limited substrate range and low diversity compared to bacteria but are effective biocatalytic agents responsible for CBM generation. For example, using a pyrosequencing and a real-time PCR approach the microbial communities in CBM gas were investigated. Results showed that the GOM_Arc_I group, with 99.3% similarity to the archaea was the dominant archaea while the most abundant and major bacterial group was the Proteobacteria with the genus *Herbaspirillum* comprising almost 76% of the sequence reads (Guo et al. 2015). Furthermore, CBM microorganisms produce methane using acetic acid, CO_2_, H_2_, methanol and other methylated compounds produced by bacteria as substrates (Akimbekov et al. 2024). Research shows that methanogens from diverse sources can be enriched and domesticated to generate CH_4_ from different coal ranks (Li et al. 2023d; Ponnudurai et al. 2022). Moreover, methanogens appear to occur more frequently and often portray as organisms of interest in subterranean coal ecosystems more often than previously anticipated (McKay et al. 2022; Meslé et al. 2013; Strąpoć et al. 2011).

Microbial consortia are therefore particularly effective biocatalysts in the bioconversion of coal to methane (Vick et al. 2019), with the four steps of biogenic CBM synthesis characterized by distinct microbial communities (Ritter et al. 2015). The hydrolytic stage of complex organic compounds is mostly facilitated by *Thielavia, Humicola, Mortierella, Trichoderma, Acremonium,* and *Alternaria* (Xianbo et al. 2020). Native bacteria like *Acetobacter* and *Pseudomonas* may break down hydrocarbons and a wide variety of aromatic chemicals (Mara et al. 2012). In addition *Bacillus* sp. encourage the hydrolysis of macromolecules (Arima et al. 1968). Hydrolysis of lipids and proteins is within *Proteiniphilum’s* capabilities (Chen and Dong 2005; Hahnke et al. 2018). During the acidogenic stage, *Clostridium, Desulfosporosinus, Petrimonas, Macellibacteroides, Tissierella,* and *Desulfitobacterium* are the primary bacteria responsible for producing the volatile fatty acids propionate, acetate, and butyrate (Xianbo et al. 2020). In acetogenesis, acetogenic bacteria including *Enterobacter, Syntrophobacter, Syntrophomonas, Sporotomaculum,* and *Desulfitobacterium* are prominent and produce acetic acid and H_2_ (Xianbo et al. 2020). Of these microbes *Syntrophobacter* can degrade propionic acid to H_2_ and acetate (Boone and Bryant 1980) while benzoate is fermented to butyrate, acetate, and CO_2_ by *Sporotomaculum* (Brauman et al. 1998). Moreover, butyric acid oxidation can be carried out by *Syntrophomonas* leading to the generation of acetic acid and H_2_ (Müller et al. 2010). *Desulfitobacterium* was found to have the capability of degrading ethanol, propionic acid, benzoic acid, and aromatic compounds (Robertson et al. 2001). Propionic acid can also be degraded by *Desulfolobus* in the presence of sulfate, whereas *Enterobacter* can generate acetic acid, formic acid, ethanol, and trace quantities of H_2_ and CO_2_ (Faiz et al. 2003; Guo et al. 2019). The diversity of methanogenic archaea in CBM is evidenced by detection and/or isolation of these microbes in formation water from different coal basins globally and include strains of the genera *Methanobacterium, Methanosarcina, Methanosaeta, Methanothermobacter, Methanolobus, Methanoculleuss*, *Methanosaeta*, *Methanolinea* and *Methanomethylovorans* which emphasizes the biotechnological potential of these species (Guo et al. 2012; Singh et al. 2012; Rathi et al. 2019; Gründger et al. 2015; Hao et al. 2021; Zhaobiao et al. 2020; Chawla et al. 2023).

## Coal biodegradation technology: applications and industrial products

**Fig. 4:**
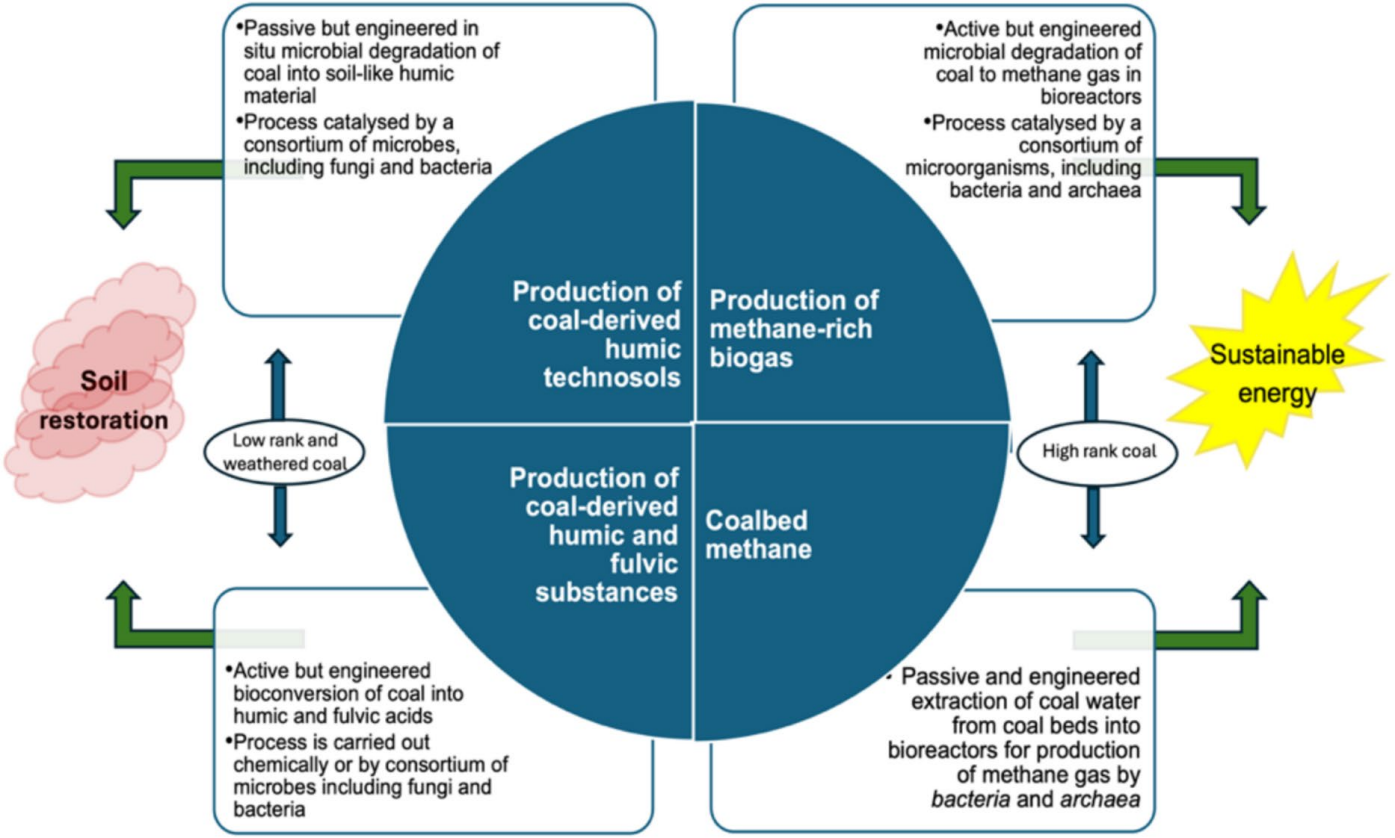
Elaboration of applications of coal biodegradation technology to upgrade the energy value of coal as a substrate for generation of methane either in situ (CBM) or in anaerobic reactors (biomethane). And the use of coal as a source of humic substances or for production of humic technosols used to increase soil organic matter

The term ‘clean coal technology’ first mentioned in 1987 (U.S. Senate Bill 911, April 1987) was intended to mean any industrial-scale process used to significantly reduce emissions of sulphur dioxide and/or nitrogen oxides during combustion of coal for electricity. In recent years this definition of clean coal technology has become somewhat blurred and is viewed by many as an oxymoron. That said, there remain countries with an abundance of coal reserve that are unable to (due to unfavourable conditions) or cannot afford to implement at scale the purportedly more ‘environmentally friendly’ energy-generating technologies such as nuclear, geothermal, hydropower and bioenergy. Such economies have little choice other than to continue with coal until circumstances allow for policy change. Consequently, and because of continued coal mining, copious quantities of waste called discard or gangue that is neither saleable nor suitable for energy generation will continue to be produced. Several clean coal possibilities exist for utilisation of low calorific brown coals and coal discard (Bezuglova et al. 2018). Included are upgrading the energy value of this material to serve as a substrate for the generation of CH_4_ or to use it as a source of humic substances or as soil amendment to increase soil organic matter, enhance soil fertility and facilitate soil restoration to support above ground vegetation. A schematic illustrating the possible applications of coal biodegradation technology to upgrade energy value for generation of CH_4_ either in-situ (CBM) or in anaerobic reactors (biomethane), source humic substances for production of technosols, and as a amendment to increase soil organic matter is shown in Fig. 4. In this section we summarise some of the recent findings that deal with the valorisation of coal and coal discard, and we review progress in bio-methanation and CBM, the use of coal for humic substance production and finally the role of coal and coal discard as a substrate in the formation of a humic-rich soil like material to support soil formation and rehabilitation of land.

Coal biodegradation depends on coal rank which is a term used to describe the geochemical changes in the parent material (i.e., woody-derived, or humic coals and, non-woody-derived or sapropelic coals) resulting from the thermal maturity of coal (O’Keefe et al. 2013). For instance, highly volatile bituminous, subbituminous, and lignite coals can be easily attacked by microorganisms due to accessible cleavage sites in the complex structure (Akimbekov et al. 2021b, a; Haider et al. 2013). Thus, many species of bacteria effectively degrade lignite and do so by synthesis of biosurfactants that enhance substrate solubility to facilitate depolymerization (Singh and Tripathi 2013; Xia et al. 2020; Shi et al. 2022). Enzymes including LiP, MnP, and LAC play a crucial role in the oxidative depolymerization of lignite (Zhao and Baker 2022; Shi et al. 2024; Chowdhary et al. 2019) whereas under anaerobic conditions, it is methanogenic bacteria that convert lignite to CH_4_. Thus, low and medium rank coal are relatively amenable to biodegradation and are ideal substrates for the extraction of humic substances and conversion to humic-rich technosols under aerobic conditions (Sekhohola and Cowan 2017). In fact, it has been reported that lignite degradation can result in a substantial amount of humic and fulvic acids (Akimbekov et al. 2020b). In contrast, bituminous coal, a higher rank coal, possesses lower oxygen content and has reduced water solubility compared to lignite, rendering it less susceptible to microbial solubilization. Nevertheless, certain microorganisms are capable of degrading bituminous coal, albeit at a slower rate (Iram et al. 2017). Even so, the depolymerization of bituminous coal is more intricate due to its higher carbonization state and more complex structure. Biodegradation of bituminous coal also yields humic substances, but the yield is typically lower than that of lignite. These humic substances have potential applications in soil enhancement and as organic fertilizers.

Highly volatile bituminous coal has a greater methane production rate when compared to medium volatile bituminous coal and anthracite (Liu et al. 2022) and in anaerobic conditions, the quantity and quality of by-products from microbial degradation differs between lignite and bituminous coal, in particular CH_4_, humic and fulvic acids (Akimbekov et al. 2020b). Thus, under anaerobic conditions microbial degradation of bituminous coal is slow, involves the breakdown of aromatic and aliphatic hydrocarbons, while gas production is generally lower than with lignite (Opara et al. 2012).

## Bio-methanation

Laboratory engineered methanogenic consortia derived from environmental samples including coal outcrops and mined waste coal (e.g., tailings and dumps) convert coal and coal waste to CH_4_ and CO_2_ (Zhang et al. 2015; He et al. 2016). These microorganisms secrete extracellular enzymes that decompose covalent bonds and other functional groups in the coal molecule to change the physical properties and facilitate its bioconversion for methane production (Xia et al. 2023). An investigation into using a two-stage microbial process for converting coal discard to methane showed that fungal solubilisation of waste coal by *Neurospora discreta* followed by microbial methanation of the solubilised products increased output 3- to sixfold (Ahmed and Sharma 2021).

There is increasing evidence to suggest that pretreatment of coal with various oxidizing agents such as KMnO_4_ and H_2_O_2_ or biological agents including LiP, MnP, and LAC, microbial EPS or associated extracellular alkalinizing agents, and biosurfactants promote the mineralization of lignite to facilitate its degradation anaerobically (Zhang et al. 2022a; Zhao and Baker 2022; Shi et al. 2024; Chowdhary et al. 2019). It is believed that the structural similarity between lignite and lignin allows lignin-degrading biocatalysts to deconstruct its complex form (Xia et al. 2024). For example, pretreatment of lignite with H_2_O_2_ enhanced degradation by microorganisms in anaerobic activated sludge via alkanes, ketones, esters, and PAH to yield CH_4_ (Zhang et al. 2024). Interestingly, the species composition of the anaerobic sludge microbiota was fungal, bacterial, and archaeal and included members of the Ascomycota, Bacteroidetes, and Methanosaeta respectively. Furthermore, the addition of biochar to anaerobic reactors enriched phenol degrading bacteria to reduce phenol content while significantly accelerating the hydrolysis-acidification process by impacting benzoylation and ring cleavage in the benzoyl-CoA pathway (Li et al. 2023a). Microbial distribution characteristics in a biogas generating system fed Shengli lignite also showed that bacterial diversity was higher than the archaea in all samples (He et al. 2020). In this system, Firmicutes were the most abundant phylum of bacteria and *Methanosarcina* was the most abundant methanogen. Microbial succession, coal bioconversion, and product formation showed that a compound flora can anaerobically digest lignite with increased methane output (Gong et al. 2023). In this study, *Cladosporium*, an aromatic-degrading fungus was dominant over *Aspergillus* and *Penicillium* which assumed this role but only after gas production.

For successful transformation of coal to CH_4_ the origin of the consortia and the composition of the microbial community are clearly important with mixed cultures seemingly the preferred candidate biocatalysts. To achieve bioconversion, many experiments have attempted to optimize protocols for increased CH_4_ output by changing substrate (i.e., coal type and/or rank), nutrient composition, employing growth stimulators, and methanogens from different underground ecosystems (Rathi et al. 2015; 2019; Susilawati et al. 2015; Barnhart et al. 2017; Davis and Gerlach 2018; Chin et al. 2024). One study found that it was feasible to enhance CH_4_ generation from lignite by stimulating the growth of native methanogens in the formation water by adding a 2-carbon alcohol (Yang et al. 2019). By doing so, these authors were able to show that ethanol caused a transition from acetoclastic (i.e., *Methanosarcina*-catalysed) to hydrogenotrophic (i.e., *Methanobacteria*-catalysed) methanogenesis.

## Coal-bed methane

Methane gas found in coal beds or seams, commonly known as CBM, is viewed as an important clean energy source, with total reserves estimated at 113–184 trillion cubic meters (T m^3^), of which 42 T m^3^ is recoverable (Al-Jubori et al. 2009; Thomas 2020). It is thus not surprising that there is a growing interest in methane-producing archaea and particularly those involved in the biogenesis of CH_4_ from CBM as a clean energy source. Produced in two ways, CBM is either (i) thermogenic; or (ii) biogenic. During the thermogenic process, the generation of CH_4_ occurs due to thermocatalytic reactions that take place during coalification, whereas the biogenesis of CH_4_ occurs through microbial utilization of high calorific C-rich sedimentary rock. Consequently, CH_4_ biogenesis is regarded as a major contributor of CBM during coal formation (Chen et al. 2023a, b; Zhang et al. 2013; Faiz and Hendry 2006). Substantial biogenic gas reserves have been reported for many countries such as U.S.A., New Zealand, and China, which constitute nearly 20% of the total regular natural gas reserves globally (Butland and Moore 2008; Green et al. 2008; Shao et al. 2018).

**Fig. 5:**
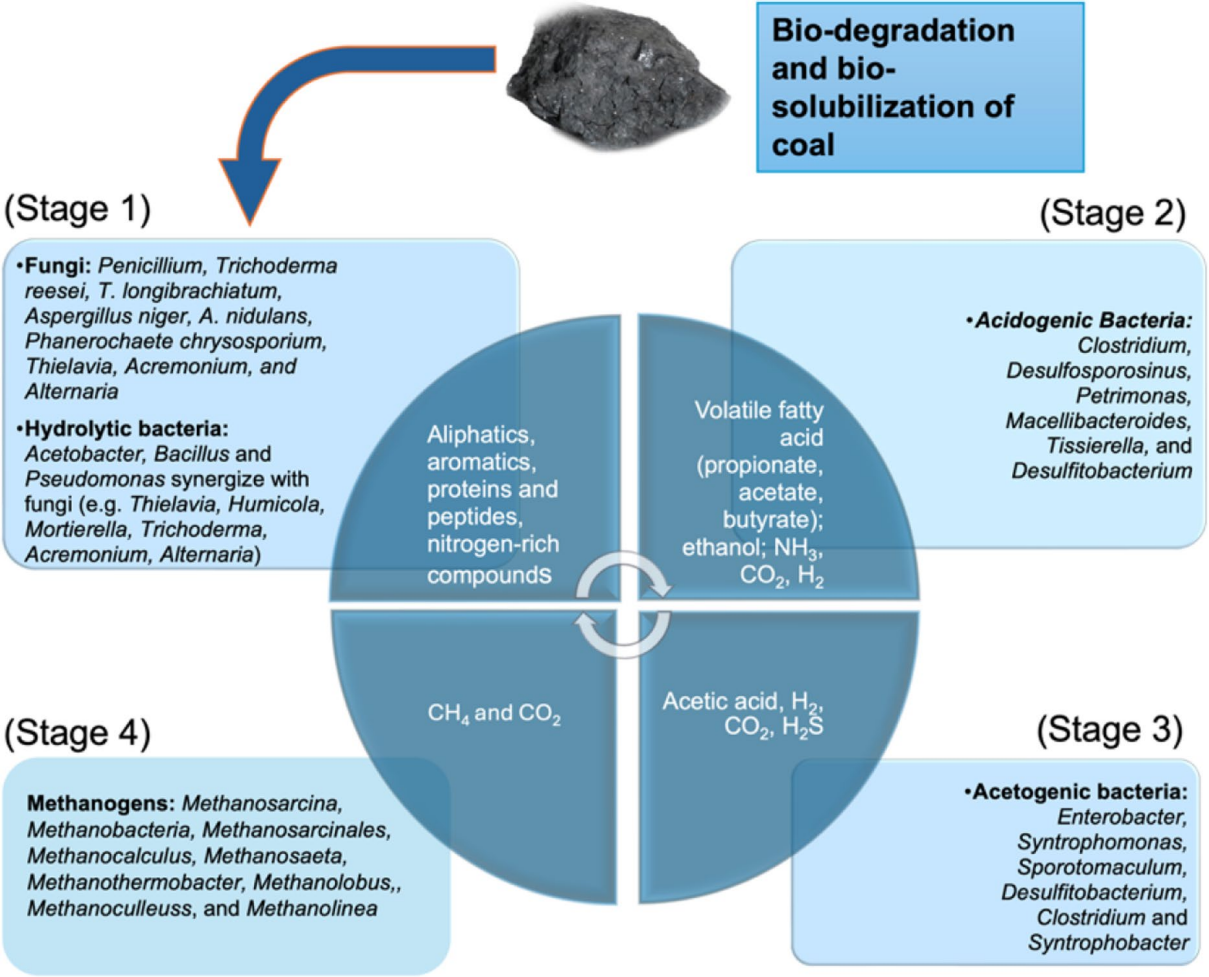
Schematic of fungal and bacterial bio-solubilization of coal and the formation of organic precursors used by methanogens to produce methane. Stage 1: distinct hydrolytic bacteria and fungi decompose coal into simpler compounds. Stage 2: acidogenic bacteria employ the hydrolysis by-products to generate organic acids, CO_2_, and H_2_. Stage 3: acetogenic bacteria primarily convert these organic acids into acetic acid, CO_2_, and H_2_S. Stage 4: methanogenesis involves synthesis of CH_4_ by a suite of methanogens either acetoclastically (from acetate) methylotrophically (from methanol or trimethylamine) or hydrogenotrophically (from H_2_ to reduce CO_2_, CO, and formate)

Subterranean coal seams are rich in fungal, bacterial, and archaeal communities that seem to operate in syntrophic association to exploit coal as a carbon source to generate various metabolites with CH_4_ and CO_2_ being the final by-products (Fig. 5). The microbial communities exist on the coal surface, in coal pores and fissures while methanogens commonly occupy the hydrosphere within and surrounding a coal seam (Li et al. 2023c). From these microbial communities, fungi and bacteria play a vital role in the degradation of lignin and other complex organics present in coal to provide a source of easily degradable carbon-containing compounds to other microbes. Methanogens utilize these metabolites, released by fungi or bacteria, and evolve CH_4_ as a product of cell growth and respiratory action. Even though microorganisms in coal seams are generally similar, they exhibit unique microbiological richness type and a dominant flora. Thus, it may be possible to speed up the carbon reduction cycle by modifying the coal seam environment in such a way that it triggers improved metabolic competence by a specific microbe or group of microbes.

Production of CH_4_ in CBM occurs primarily in the adsorbed state on the porous surface of coal particles. With a reduction in pore size, molecules of CH_4_ change from an adsorbed state to a free form. Concomitant with the extraction of billions of tons of coal over the past decades and on an annual basis, huge amounts of CBM have been released to atmosphere, which has contributed massively to global greenhouse gas emissions and associated effects. Thus, the significance of CBM removal is becoming increasingly important, and improved capturing is encouraged to increase the efficiency of the mining process and utilization of coal resources, but also for the retrieval and consumption of CBM as a cleaner energy source (Liang et al. 2021).

The biogenic CBM process as expected typically involves (i) hydrolysis; (ii) acidogenesis; (iii) acetogenesis; and (iv) methanogenesis. Hydrolysis is the rate limiting step in the anaerobic digestion of complex organic matter. This phase is facilitated by the action of extracellular hydrolases produced by hydrolytic bacteria. And the rate of substrate hydrolysis is affected by the quality and chemical composition along with process parameters such as pH, temperature, and concentration of solids (Sträuber et al. 2012). Compounds like alkanes, simple aromatics, phenolics and fatty acids are characteristic products of coal bioconversion by hydrolysis in anaerobic environs. Acidogenesis follows and is typified by utilization of the products of hydrolysis and conversion of these into VFA (i.e., propionic acid, acetic acid, butyric acid, isobutyric acid, and valeric acid), H_2_, CO_2_ and alcohols. Thereafter, products of acidogenesis such as VFA and alcohols can be converted into acetate via the activities of enzymes produced by acetogenic bacteria (Arelli et al. 2022). Further biodegradation of the above intermediates occurs by secondary fermentative bacteria, commonly known as syntrophic bacteria, leading to the generation of acetic acid, H_2_, CO_2_, and methanol for methanogens (Thauer et al. 2010). The final stage methanogenesis, plays a vital role in determining process efficiency which appears to require a supply of acetic acid, CO_2_ and methylated compounds (Kurth et al. 2020).

## Coal-derived humic substances

Coal that has high ash content and other impurities has restricted use for energy generation. However, high availability and low price per ton are indeed motivators for beneficiation of the material to humic substances and for these to be used in chemistry and agriculture (KluIáková and Pavlíková 2017; De Souza and Braganc 2018; Akimbekov et al. 2020b; Gong et al. 2020; Jeong et al. 2021; Yang et al. 2021). For example, lignite is desired for development as a feedstock for value-added chemicals and products (Wang et al. 2017a; Perminova 2019). Such value-added products include geopolymers (Bankowski et al. 2006; Mucsi et al. 2014; Yi et al. 2018), metal-stabilized humic substance nanoparticles (Wang et al. 2017a) and other nanostructures (Lipczynska-Kochany 2018; Sachkova et al. 2019). Following development of methods for the extraction of humics from coal, several commercial coal-derived humate products have appeared in the market (Lobartini et al. 1992; Akimbekov et al. 2020b; 2022).                                                                                 

Furthermore, chemical treatment of coal to enhance humic acid yield can lead to improved product quality (Fatima et al. 2021). The economic value of coal products such as fuels and chemical feedstocks, derived through conventional techno-chemical methods is influenced by energy content, production cost, and environmental impact (Yan et al. 2022; Zeng et al. 2019). For example, products obtained from coal through direct and indirect liquefaction exhibit distinct techno-economic outcomes (Gao et al. 2018; Xie et al. 2018). Indirect liquefaction uses more water, consumes more energy, and generates greater emissions compared to the direct method (Zhou et al. 2017). Even so, of greater value to soil scientists and rehabilitation technologists is the by-product coal char, which can be used as a soil amendment to improve soil health and fertility (Amoah-Antwi et al. 2020; Panday et al. 2021; Cooper et al. 2022; Thapa et al. 2024). It offers benefits like increased water and nutrient retention, improved soil structure, and enhanced plant growth (Cooper et al. 2022). Coal char can also help reduce nutrient leaching and greenhouse gas emissions from soil (Panday et al. 2020; Cooper et al. 2022). Studies suggest that coal char reduces CO_2_ emissions from soil, but effects on greenhouse gas (GHG) emissions are variable and depend largely on soil type and prevailing conditions. Most recently, Bai et al. (2025) determined, by meta-analysis of 2594 paired observations from 157 studies conducted between 2000 and 2024, that the addition of ‘char’ to soils led to a 13.1 % decrease in N_2_O emissions and that the associated increase in microbial biomass contributed to a reduction in CO_2_ and CH_4_ emissions.

Humic substances occur naturally in soils and are reported to benefit soils through mitigation of compaction, promoting microbial species richness and abundance, and by enhancing nutrient uptake by plants (Piccolo et al. 1997). However, the pathway by which humics arise from coal has yet to be elucidated. The in-situ bioconversion of various coals and waste coals to humic and fulvic acids provides an opportunity to study the details that underpin this bioconversion process.

In the early 1960s, the potential of low-grade coal as material for use as a soil amendment was recognised (Giannouli et al. 2009). Thus, Leonardite, weathered coal, lignites and other brown coals are frequently used as substrates to prepare humic substance-rich extracts using alkali or acid hydrolysis. Nonetheless, coal rank is not often considered although it may play a crucial role in the activity of this biostimulant (Kurniati et al. 2018; Zhou et al. 2019a, b; Cheng et al. 2019). It has been established that soil-localised microbial bioconversion of coal (Sekhohola-Dlamini et al. 2022; Akimbekov et al. 2022) results in better soil quality by enhancing soil organic carbon content (Valero et al. 2016). Furthermore, several strains of coal biosolubilising microorganisms mimic the suite of characters common to plant growth-promoting rhizobacteria (Titilawo et al. 2020). However, since humic substance extraction rate by microbial degradation is very low in nature, artificial humification processes such as aerobic composting and hydrothermal treatment have attracted a great deal of attention as the most important strategies in humic substances production including formation of humic acid, fulvic acid, and humin which are most promising for soil remediation (Hu et al. 2022). In addition, humification represents a promising strategy for organic pollutant removal. Progress in synthesizing artificial humic substances has made directed humification of recalcitrant organic pollutants possible. Thus, it is posited that both biotic and abiotic degradation and humification processes coexist; mechanisms responsible for biotic and abiotic humification remain largely unknown; and whether an organic forms humics depends very much on the aromaticity of the molecule and its susceptibility to microbial interaction (Wang et al. 2022b).

Early work by Haider and Martin (1967) showed that cultures of the ascomycete *Epicocium nigrum* produced humic acid in vitro, which was evidenced by the formation of polymers and disappearance of substrate phenols. A more recent study, using the lignite-depolymerizing bacterium *Pseudomonas cepacia* DLC-07, revealed that this bacterium first depolymerized and thereafter polymerized lignin-like substrates into larger polymeric compounds (Crawford and Gupta 1991a; 1991b). Thus, polymerization reactions predominate when substrates are rich in lignin-type compounds. Since microorganisms synthesize aromatics and exude these into the soil environment (Zwetsloot et al. 2018; 2020) and the atmosphere (Misztal et al. 2015), microorganism-polymerized aromatics would seem to contribute to the formation of humic substances and presumably by a mechanism like that elegantly elaborated by Sekhohola-Dlamini et al. (2022). Similarly Kallenbach et al. (2016) proposed that accumulation of humic substances was due to microbial community activity, and that the greater the microbial abundance of a soil the more organic matter would be accumulated in that soil. The contemporary view on humic substances as opined by Baveye and Wander (2019) in their perspective together with the information above, therefore corresponds closely to the ideas originally posited by Waksman (1936).

In addition to the hormone-like effects of humic substances summarised by Sekhohola-Dlamini et al. (2022) and its well-known bioactivity (Zhao et al. 2023), research has revealed that humics also impact expression of the senescence dependent genes *CYP*, *NAC2* and *LOX* to mitigate senescence-induced damage and strengthen antioxidant systems through induction anti-aging pathways (Ghafari Rahbar et al. 2022). In the soil environment, humic substances act as natural electron shuttles that potentially facilitate chelation of nutrients and pollutants into organo-mineral fertilisers containing macro and micronutrient metals e.g., lignite-extracted humic acid-metal complexes containing Fe, Mg, or Zn that increase soil humic content and the availability of essential nutrients to enhance substrate fertility (Iqbal and Aftab 2023). Indeed, lignite-derived humic substances improved soil organic matter, edaphic factors, and maize yield and, at the same time, served as a waste disposal mechanism (Solek-Podwika et al. 2023; Sarlaki et al. 2024). Engineered nitro-humic fertilizer (NHF), synthesized using ozone oxidation and nitrogen enrichment increased soil water holding capacity, extended the water-retention period and reduced soil urease enzyme activity (Sarlaki et al. 2023) while a slow release humic acid urea fertilizer (HA-N) increased storage root growth and yield (Chen et al. 2017) and the lignite-induced increase in humic acid content of compost was achieved by improving LAC activity and microbial transformation of humics (Wang et al. 2024). And lignite-converted bioorganic fertilizer (LCB) was shown to improve soil edaphic factors (Chen et al. 2023a, b). Thus, microbial abundance, robustness, and positive cohesiveness of members of the Gammaproteobacteria, Gemmatimonadetes, and Methylomirabilia, and the fungi of the class Glomeromycota and the clade GS13, increased substantially while vulnerability of bacterial cooccurrence networks was reduced to enhance the stability of the soil bacterial community. Similarly, analysis of temporal changes in the sequence of phytoremediation of lignite mine tailings showed that the bacterial population was dominated by members of the Firmicutes (32%-72%), Bacteroidetes (1%-10%) and Proteobacteria (1%-24%) and that the initiation of primary microbial succession occurred coincident with revegetation (Singh et al. 2022b).

## Coal-derived humic technosols: soil restoration and land rehabilitation

Coal is thought of as a recalcitrant material and perceived as naturally toxic. Thus, application of low-calorific waste coals as soil amendment was initially considered unsafe. However, many different types of low rank coals are more and more being used to produce improved technogenic soils or technosols (Tripathi et al. 2016; Amoah-Antwi et al. 2020; Sekhohola-Dlamini et al. 2022). Without continual replenishment, soils become depleted of essential nutrients and organic carbon, and some form of amendment is required to maintain soil fertility and productivity. Thus, the use of weathered coal in soil reconstruction has become crucial in restoration and rehabilitation of land affected by mining and in particular, open cast coal mining. In addition to in situ coal bioprocessing (Sekhohola-Dlamini et al. 2022; Sarlaki et al. 2024), the application of digestate, the liquid effluent from anaerobic bioreactors containing coal inoculated with microbial enrichments when compared to organic fertilizers, stimulated plant growth on soils amended with this product (Fallgren et al. 2021). This, and related findings, indicate that coal-derived products are intimately involved in promoting an increase in soil organic matter and supporting plant growth. Also, coal is rich in humic substances which when liberated either chemically or biologically, can be formulated as a soil amendment and used in agriculture, horticulture and to support phytoremediation (Du et al. 2020; Sekhohola-Dlamini et al. 2022). Thus, addition of humic extracts, rather than coal itself, is considered more efficient as humics are more easily accessible to soil mineralisation processes. In a comparative greenhouse study by Akimbekov et al. (2020a, b), one set of sandy loam soil was treated with Leonardite (a vitreous mineraloid formed by the oxidation of lignite) while the other was supplied Leonardite-derived humics. Substrate with added humics had higher bacterial diversity and elevated plant production measured as crop yield (Akimbekov et al. 2020b). The lower bacterial diversity and crop yield in Leonardite-amended substrate may indicate delayed release of humics or suppression by leaching and/or weathering of inhibitors.

Technosols are artificial soil-like substrates containing lime and composed of organic materials including coal, solid waste, compost, industrial and agricultural waste that help to improve the physicochemical and biological properties of the amended substrate (Modarres et al. 2015; Weiler et al. 2020; Firpo et al. 2021; Sarlaki et al. 2024). Generally, undisturbed and healthy soils in mesic environments typically have taxonomically and functionally diverse microbial populations that mineralize organic substrates and release humic and other plant growth promoting substances into soils while maintaining nutrient cycling (Bardgett and van-der-Putten 2014) and biodegrade pollutants such as PAH and coal (Valero et al. 2014; Malik et al. 2016; Baylon et al. 2017; Nsa et al. 2021; Ren et al. 2023). It is the latter that appears to help increase humics release from recalcitrant materials. Several studies have exploited this phenomenon by using microbes with coal-degrading capabilities (Cubillos‑Hinojosa et al. 2015; Valero et al. 2015; Sekhohola and Cowan 2017; Filho et al. 2020) together with various mine spoils including coal mine spoil. These soil-like artefacts used as the basis of technosols are defined as solid or liquid substances created or substantially modified by humans as part of the industrial process or brought to the surface and deposited in an environment where they do not commonly occur (Schad 2018).

Traditionally, in many regions rehabilitation of land disturbed by coal mining relies on the use of topsoil to establish plant cover. This approach allows for the underlying pollutant material to be “naturally” weathered and slowly degraded and transformed as a result of in situ microbial activity. And when left to nature, rehabilitation may take many years, and a successful outcome cannot be guaranteed. Inoculating such land with carbonaceous substrates and coal degrading microorganisms is expected to increase the effectiveness of the rehabilitation process through promoting in situ biodegradation of the coal material into a product that supports rapid and successful establishment of plant cover. This phenomenon was demonstrated in both the laboratory and at commercial scale. First, a controlled pot trial experiment revealed that biodegradation of low-grade coal in the presence of coal degrading fungal suspension of 10 ml and arbuscular mycorrhizal fungi (10 g in 2 kg of substrate) led to the formation of a humic-rich soil-like material within the *Cynodon dactylon*/coal rhizosphere (Sekhohola and Cowan 2017). Results also showed that an increase in humic substances concentration in the growth media of *Cynodon dactylon* correlated positively with the accumulation of above ground biomass (Sekhohola and Cowan 2017). Commercial scale field trials confirmed these findings and showed that in situ inoculation of coal dumps with coal-degrading and mycorrhizal fungi followed by seeding, using a selection of annual and perennial grass species, was viable and sustainable for commercial rehabilitation (Cowan et al. 2016).

Waste coal dumps aside, open cast mining of coal leaves large gaping wounds in the soil that are backfilled with overburden and spoil as part of an intensive land reclamation process. Restoring the function of such barren soils depends on restoring the soil microflora and fauna and ensuring the presence of sufficient organic matter to improve the overall soil physical, chemical and biological properties. Here, several studies indicate that the in situ bacterial and fungal degradation of coal results in formation of a soil-like humic-rich substrate that can function as an amendment to support (re)vegetation (Sekhohola and Cowan 2017; Ivanova et al. 2024). In a pot trial, Wang et al. (2024) investigated the feasibility of using coal gangue inoculated with 3 ml overnight culture of *Stenotrophomonas bentonitica* BII-R7 in 480 g sandy soil to improve its productivity. Their results showed comparatively better alfalfa seed germination and seedling establishment after inoculation of the growth media and revealed that this bacterium also had plant growth promoting effects (Wang et al. 2024). Titilawo et al. (2020) also reported that coal-degrading bacterial isolates exhibited traits characteristic of plant growth promoting microorganisms. Despite studies like these, and others (Sarlaki et al. 2024; Rahayu et al. 2024), coupled with evidence to support the idea that microbial treatment of coal can be used to improve the quality of depleted soils to facilitate reclamation of land disturbed by mining, application of the coal biodegradation technology at scale remains underexplored.

Furthermore, to improve soil quality and facilitate land restoration, specific microorganisms are introduced into degraded soils through techniques that ensure their survival, establishment, and functional activity. The selection of microbial strains is based on functional characteristics, such as nitrogen fixation, phosphate solubilization, organic matter decomposition, or resistance to abiotic stress (Khan et al. 2018; Titilawo et al. 2020; Masudi et al. 2024). These microorganisms are often incorporated into suitable carriers, such as peat, vermiculite, compost, or liquid suspensions, to enhance their viability and ease of application. Inoculation methods include seed coating, soil drenching, root dipping, or mixing with organic amendments, depending on the site conditions and the targeted vegetation. Following application, factors such as soil moisture, temperature, and pH are regulated to promote microbial colonization and activity. Collectively, these strategies contribute to restoring soil fertility, enhancing soil structure, and improving the overall functionality of ecosystems in reclaimed areas (Iqbal et al. 2025). And under appropriate (experimental) conditions it should be possible to determine the kinetic parameters and suite of intermediates formed in the bioconversation of a carbonaceous substrate like coal into the humic and fulvic acids contained within the technosol. Focus on the latter will undoubtedly contribute to closing the knowledge gap that currently exists in sense of the passive, in situ biodegradation and solubilization of coal.

## Summary and prospects

Coal biodegradation is critical in combating the many detrimental impacts of disposed coal mine waste, which are both long and short term, and must be incorporated into the development of diversified production technologies for both economic and ecological reasons. On one hand coal mining remains a significant contributor to many global economies, and on the other, coal mining places unnecessary strain on ecologically sensitive areas, by increasing pollution and ecological destabilization potential. Eventual environmental degradation is evidenced by loss of resources and the destructions of indigenous flora and fauna. Additionally, the move by some economies away from coal combustion has increased exploration of alternative uses for coal, including clean energy derivation (e.g., CBM and biomethanation) and for the extraction of humics for use in soil restoration and land rehabilitation. Thus, the motivation to compile a review outlining the current state-of-the-art of coal biodegradation and biosolubilization. In part, this stems from the fact that there is an urgent need to balance mineral extraction with sustainability and management of coal waste is an important aspect. There are various schools of thought regarding sustainable coal mining and handling of coal mine waste, many of which are informed by concerns including the ever-present environmental impact of continued coal extraction and utilization and the accumulation of waste. Several interventions have been explored towards eradication of the long-standing legacy of coal mining waste, however, integration of biological technologies driven by microbial solubilisation and degradation of coal is still lacking. Consequently, this work is also motivated by the possible contribution this paper can make in assembling empirical data on the biocatalysts and mechanisms that underpin the biological degradation and solubilization of coal, and the untapped opportunity to produce new bioprocess technologies and value-added by-products.

In reviewing the current literature on the biological degradation and solubilization of coal it becomes apparent that research continues to identify microorganisms capable of degrading and solubilising coal from both aerobic and anaerobic environments. Unfortunately, effort to elucidate the pathway from parent macromolecular coal to the respiratory end-product gases CO_2_ and CH_4_ is largely absent or at best, fragmentary. Aside from humic substances which are formed as a by-product of the biodegradation process, very few other key intermediates in the process have been identified and their further metabolism remains obscure. It is also interesting that while coal bioprocessing can be either aerobic or anaerobic, the main value-added by-product remains humic substances (with CO_2_ and CH_4_ as microbial respiration products). And the aerobic biodegradation of coal follows a series of oxidative depolymerization and hydrolytic reactions catalyzed by oxidizing agents and oxidative enzymes and hydrolases, the anaerobic process follows a sequence of four stages in which the initial stage, a critical step in the process, is hydrolysis. Thus, in both aerobic and anaerobic processing hydrolysis destabilizes the polyaromatic structure of coal for subsequent stages that culminate in release of CO_2_ (and likely some CH_4_) from aerobic reactions and CH_4_ and CO_2_ from the anaerobic reactions. Catalysis of aerobic reactions is predominantly by fungi and bacteria, while the archaea are largely responsible for anaerobic bio-processing of coal to CH_4_.

At commercial scale, the coal biodegradation technology has mainly been applied in the rehabilitation of waste coal dumps. Successful application of in situ coal degradation through direct inoculation with a microbial consortium has been demonstrated on South African waste coal dumps with a technology termed Fungcoal. Such demonstration has profound implications on the traditional ways of handling waste coal dumps, which have long been exclusively designed from an engineering perspective that focuses on compacting and watering the dumps prior to soil application to promote plant growth. It has been reiterated that microbial inoculation is essential to steer soil restoration and overcome the naturally slow restoration of degraded terrestrial ecosystems, which is often unsuccessful due to a number of abiotic factors and hostile soil conditions that impede microbial proliferation. Thus application of coal biodegradation technology as an in situ rehabilitation strategy can promote large scale use and consumption of waste coal that would otherwise be discarded. Production of humic substances has been extensively researched and upscaled to commercial scale with many products used for soil amendment currently available on the market. Methane generation from coal either by bio-methanation or through CBM are additional process technologies that have the potential to convert various forms of coal, including waste coal  to a value-added product and, at scale. Evidence from studies that have evaluated the upscaling dynamics of this process under bioreactor conditions is emerging and so far, the results have shown feasibility for industrial production of biogas.

## Conclusions

Despite the information presented above and the limited small scale successes, there is no empirical evidence of in-depth elucidation of the microbial genome that functionally drives either the aerobic or anaerobic processes of coal biodegradation. Consequently, there remains a clear knowledge gap on specific enzymes that catalyze the anaerobic processes, the products formed, and the sequential identification of bacterial species involved in each of the four stages of the anaerobic process is also lacking. Thus, future research should begin with targeted functional genomics of the most promising coal‑degrading enzymes such as LAC, LiP, and esterases potentially by heterologous expression and CRISPR knockouts to quantify the enzymatic roles and kinetic parameters of these catalysts. Simultaneously, time‑resolved transcriptomics of various fungal-bacterial consortia under controlled oxygen gradients may reveal regulatory switches between solubilization, assimilation and methanogenesis. Data therefrom, should feed into genome‑scale metabolic models of dominant consortium members, integrating multi‑omics inputs to predict bottlenecks and refine reactor and/or bioprocess conditions. Finally, the most promising genetically or process‑optimized strains and consortia must be validated in pilot‑scale (100–1000 L) reactors over multiple batches and continuous cycles, measuring degradation rates, product titres, and long‑term stability to bridge the gap toward commercial coal bioprocessing. In addition, interdisciplinary collaboration will be key to advancing coal bioprocessing: materials scientists can design novel support matrices to enhance microbial attachment and mass transfer, environmental and chemical engineers can develop predictive reactor and transport models for scale‑up, data scientists can implement real‑time monitoring and control algorithms, and agronomists can validate soil‑amendment performance in field trials. By integrating these diverse disciplines, technical barriers can be overcome and the translation of coal‑based biotechnologies from the lab to industry accelerated.

## Data Availability

Availability of data and materials Datasets generated and/or analysed during the study are available from the corresponding author on reasonable request.
